# Comprehensive interrogation of synthetic lethality in the DNA damage response

**DOI:** 10.1038/s41586-025-08815-4

**Published:** 2025-04-09

**Authors:** John Fielden, Sebastian M. Siegner, Danielle N. Gallagher, Markus S. Schröder, Maria Rosaria Dello Stritto, Simon Lam, Lena Kobel, Moritz F. Schlapansky, Stephen P. Jackson, Petr Cejka, Marco Jost, Jacob E. Corn

**Affiliations:** 1 Institute of Molecular Health Sciences, Department of Biology, Swiss Federal Institute of Technology (ETH) Zurich, Zurich, Switzerland; 2 Institute for Research in Biomedicine, Faculty of Biomedical Sciences, Università della Svizzera italiana (USI), Bellinzona, Switzerland; 3 Cancer Research UK Cambridge Institute, University of Cambridge, Cambridge, UK; 4 Gurdon Institute and Department of Biochemistry, University of Cambridge, Cambridge, UK; 5 Department of Microbiology, Harvard Medical School, Boston, MA USA

**Keywords:** Double-strand DNA breaks, DNA damage response

## Abstract

The DNA damage response (DDR) is a multifaceted network of pathways that preserves genome stability^1,2^. Unravelling the complementary interplay between these pathways remains a challenge^3,4^. Here we used CRISPR interference (CRISPRi) screening to comprehensively map the genetic interactions required for survival during normal human cell homeostasis across all core DDR genes. We captured known interactions and discovered myriad new connections that are available online. We defined the molecular mechanism of two of the strongest interactions. First, we found that WDR48 works with USP1 to restrain PCNA degradation in FEN1/LIG1-deficient cells. Second, we found that SMARCAL1 and FANCM directly unwind TA-rich DNA cruciforms, preventing catastrophic chromosome breakage by the ERCC1–ERCC4 complex. Our data yield fundamental insights into genome maintenance, provide a springboard for mechanistic investigations into new connections between DDR factors and pinpoint synthetic vulnerabilities that could be exploited in cancer therapy.

## Abstract (web summary)

Combinatorial CRISPRi screening was used to map genetic interactions in DNA damage response pathways, revealing known and new connections, including the roles of WDR48 and USP1 in preventing under-replication and SMARCAL1 and FANCM in remodelling persistent cruciform DNA structures.

## Main

Numerous DNA repair pathways, collectively known as the DNA damage response (DDR), counteract diverse threats to genome integrity^1,2^. A failure to maintain genome integrity can lead to human diseases, such as premature ageing and cancer^1^. Even during unperturbed proliferation, the DDR is activated to deal with issues ranging from mismatches to complete DNA replication fork collapse. The roles of many individual DDR pathways have been resolved in detail^1,2^. However, the DDR is buffered by factors with overlapping functions and pathways that compensate for each other despite completely distinct molecular mechanisms. This can mask gene functions in essential DNA repair processes and necessitates a systematic dissection of genetic interactions in the DDR.

Mapping genetic interactions can reveal new and unanticipated biological insights^4^. Synthetic lethality, when cells tolerate the loss of individual genes but not their simultaneous inactivation, suggests buffering through backup pathways. Genetic interactions may also suggest routes to precision therapies, such as when DDR mutant cancers become hyper-dependent on backup DDR pathways for their survival.

To comprehensively map genetic interactions in the DDR, we turned to CRISPR interference (CRISPRi) dual-guide screening^4^. This approach enables the simultaneous expression of two single guide RNAs (sgRNAs) to robustly silence the expression of two defined genes and has been used to map genetic interactions between selected pathway subsets and assign new functions to poorly characterized genes^4^. We developed a new DDR-centric dual-guide library called SPIDR (Systematic Profiling of Interactions in DNA Repair) and used it to map approximately 700,000 guide-level and 150,000 gene-level interactions for 548 core DDR genes. We deployed the SPIDR library in unperturbed and karyotypically normal human retinal pigment epithelial 1 (RPE-1) cells, which have no known deleterious mutations in key DDR genes (Supplementary Table 1). This approach systematically discovered new aspects of DDR biology required during normal homeostasis, as well as therapeutically relevant synthetic lethal relationships.

Mechanistic interrogation revealed two new functional relationships between WDR48–USP1 and LIG1 or FEN1 (LIG1/FEN1) and between FANCM and SMARCAL1. First, *WDR48*–*USP1*:*LIG1*/*FEN1* synthetic lethality is driven by unrestricted RAD18-mediated PCNA ubiquitylation. This leads to PCNA degradation, with ensuing DNA under-replication and genome instability. Second, we found that the translocase activities of FANCM and SMARCAL1 counteract cruciform DNA structures at TA-rich repeats in late replicating regions. Cruciforms are prone to arise during normal DNA replication^5,6^, and without FANCM and SMARCAL1, they persist and are susceptible to cleavage by the ERCC1–ERCC4 complex^7^.

A web-based interface to the complete DDR interaction dataset is available at https://spidrweb.org. Altogether, our study revealed fundamental insights into how genome stability is maintained and provides a starting point for mechanistic resolution of DDR pathways and the identification of new targets for cancer therapy.

## A map of genetic interactions in the human DDR

To systematically map genetic interactions in the DDR, we designed a combinatorial CRISPRi library targeting all genes with the gene ontology term ‘DNA repair’ (GO:0006281; 548 genes). We performed a screen in the absence of exogenous DNA damage to query interactions between pathways that are required for normal cell homeostasis. We opted for a CRISPRi rather than CRISPR nuclease screen to avoid generating double-strand breaks (DSBs), a source of DNA damage that could activate the DDR. By not inducing complete loss of function of target genes, we reasoned that CRISPRi would also enable us to identify genetic interactions involving essential DNA repair genes and to better model DDR deficiencies found in cancer, which frequently arise from hypomorphic mutations or epigenetic silencing resulting in reduced expression^8,9^.

**Fig. 1:**
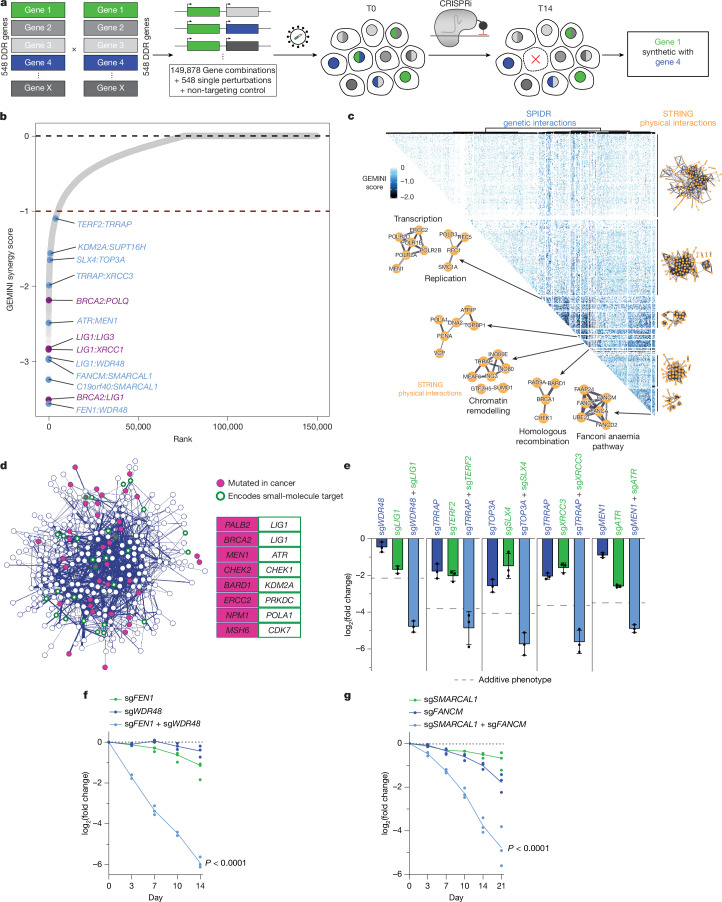
Comprehensive combinatorial screening identifies synthetic relationships between DDR factors. **a**, Guide RNAs targeting 548 core DDR genes (GO:0006281) are systematically combined into a dual-guide lentiviral library termed SPIDR. Guide RNA samples were taken at time point zero (T0) and day 14 (T14). The screen was performed once in biological duplicates. **b**, Rank-ordering all pairwise interactions by sensitive GEMINI score identifies known synthetic relationships (magenta) and nominates new candidates (light blue). Red dashed line indicates a sensitive GEMINI score of −1. **c**, Clustered heat map of SPIDR genetic interaction profiles. Clusters with similar sets of genetic interactions encompass STRING annotated physically interacting sub-complexes. **d**, Synthetic relationships filtered at a GEMINI score of −1 or less are represented as a complex network, with the strength of the interaction determining the width of the edge. Nodes are coloured on the basis of whether they are mutated in cancer (magenta filled; COSMIC tier 1 and tier 2 genes), small-molecule targets (green rings; DGIdb targets) or both (magenta filled and green rings). Selected genetic interactions that may be therapeutically actionable (one member mutated in cancer and the other targeted by an existing small molecule) are highlighted. **e**, Experimental validation of five new synthetic lethal interactions identified by the SPIDR screen. Experiments were performed in triplicate, and the percentage of each cell population was quantified by flow cytometry. The log_2_ fold changes on day 14 relative to day 0 are shown. The additive phenotype (dashed line) was calculated by summing the individual depletion phenotypes. Error bars represent mean ± s.d. Complete survival time courses are shown in Extended Data Fig. 3c–h. **f**,**g**, Dual-colour flow cytometry survival time course of the *FEN1*:*WDR48* (**f**) and *FANCM*:*SMARCAL1* (**g**) interaction. Lines represent mean of three technical replicates. *P* values were calculated using two-way analysis of variance (ANOVA) between cells expressing both sgRNAs and each sgRNA alone. Source data

Initial sgRNAs targeting each gene were based on the human CRISPRi-v2 library^10^. The final library consisted of at least two sgRNAs targeting each gene, each paired with every other sgRNA (Fig. 1a). For each sgRNA targeting an essential gene, we included an extra mismatched variant sgRNA that was empirically validated or predicted to confer partial knockdown^11^. Each targeting sgRNA was paired with 15 non-targeting sgRNAs, and 225 non-targeting-only dual sgRNA pairs were included as negative controls. In total, the library queries 697,233 guide-level interactions and 149,878 gene-level interactions (Supplementary Table 2). Each sgRNA combination was uniquely synthesized on an oligonucleotide chip and cloned into a dual-sgRNA lentiviral expression vector^12^. For ease of reference, we called this library SPIDR.

**Extended Data Fig. 1:**
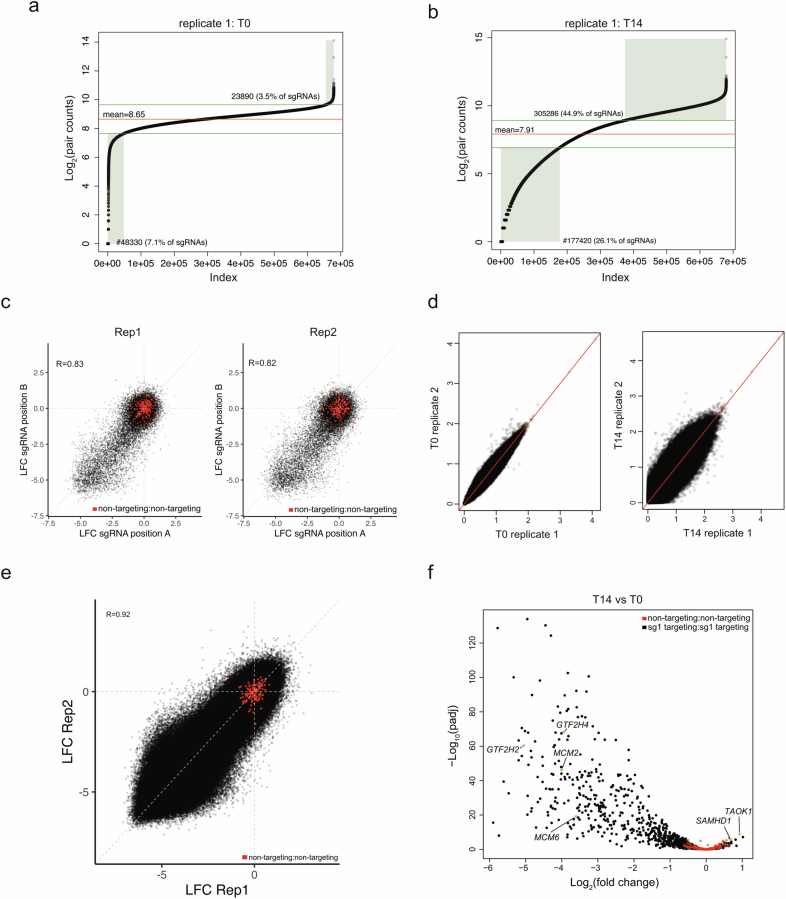
General Quality control of SPIDR screen. Quantification of log_2_ pair count of replicate 1 at **a**, T0 and **b**, T14 shows high coverage at T0 and drop out of many combinations at T14. **c**, LFC comparison between library elements (targeting:non-targeting) with the targeting sgRNA in library position A or B in screen replicate 1 and 2. **d**, log(RPM + 1) analysis of screen replicate count data at T0 and T14. **e**, LFC comparison T14vsT0 for all library elements. **f**, Volcano plot of log_2_ fold change of non-targeting (non-targeting:non-targeting) and single gene (targeting:targeting) targeting library elements at T14 compared to T0. Common essential genes (e.g., *MCM2*, *GTF2H2*) show high negative values while tumor suppressors *SAMHD1* and *TAOK1* show a positive log_2_ fold change, respectively. Statistical significance was determined using the Wald test, with Benjamini-Hochberg post-test.

Using the SPIDR library, we performed a growth-based screen in RPE-1 *TP53* knockout (KO) cells, which have an otherwise intact DDR genetic background. We generated a clonal cell line stably expressing catalytically inactive Cas9 fused to a Krüppel associated box (KRAB) transcriptional repressor domain^13^. Cells were transduced with the lentiviral library, and time point 0 (T0) cells were collected 96 h after transduction. The final time point was collected 14 days later (T14) (Fig. 1a). Next-generation sequencing and sgRNA quantification identified sgRNA pairs whose knockdown inhibited cell proliferation (Extended Data Fig. 1a,b).

We confirmed that the SPIDR library performed well by comparing screen replicates, ensuring equal performance of the guide RNAs in either the first or second position and confirming that perfectly matched guide RNAs targeting essential genes were strongly depleted (Extended Data Fig. 1a–f). All metrics indicated the high technical performance of the screen and the ability to reliably capture individual gene phenotypes.

**Extended Data Fig. 2:**
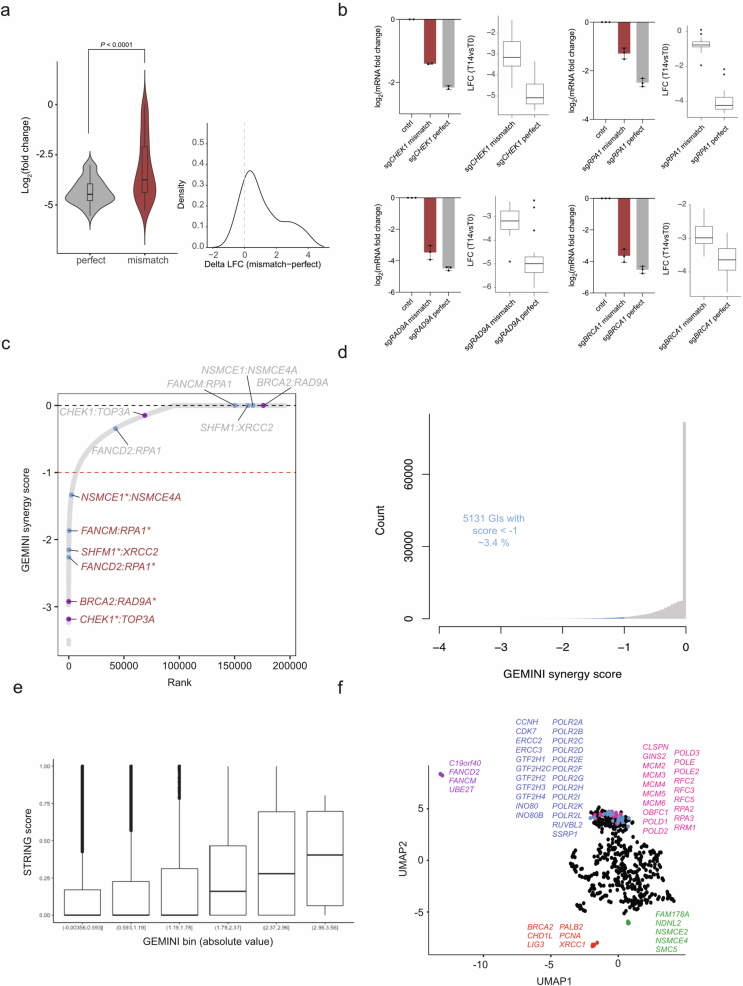
Mismatch guide analysis and correlation of SPIDR interactions with physical interactions. **a**, Growth phenotypes of mismatched sgRNAs for strongly essential genes. Violin plot showing the log_2_ fold change of 91 essential sgRNAs (LFC ≤ −3) and their mismatched variant. Mismatched variants have significantly decreased growth phenotype, two-tailed Wilcoxon rank sum test. Density plot for essential sgRNAs showing delta LFC for mismatched subtracted by perfect sgRNAs. Box plots show median values and data within the 25th–75th percentile, with whiskers representing the upper and lower percentiles +/− 1.5 * interquartile range. Most mismatched variants reduce the negative growth phenotype, leading to the majority of delta LFC density > 0. Vertical dashed line indicates delta LFC of 0. **b**, log_2_(mRNA fold change) measured by RT-qPCR of perfect and mismatch sgRNAs for selected essential genes. Error bars represent mean ± s.d. RPA1, RAD9A, and BRCA1 (*n* = 3 experimental replicates) and CHEK1 (*n* = 2 experimental replicates). LFCs of the selected sgRNAs (targeting:non-targeting) from the SPIDR screen (right). The boxplot formats are the same as in **a**. **c**, Sensitive GEMINI score rank-ordering of all binary interactions with perfect sgRNAs or mismatched sgRNAs (asterisk) for essential genes. Mismatch sgRNAs help to recover known (magenta) as well as novel (light blue) genetic interactions. **d**, Histogram of GEMINI synergy scores of all genetic interactions. **e**, Sensitive GEMINI scores from the SPIDR library were separated into six equal bins and compared to STRING probability scores retrieved from version 12 of the human database^78^. A stronger SPIDR genetic interaction correlates with STRING score. Box plots show median values and data within the 25th–75th percentile, with whiskers representing the upper and lower percentiles +/− 1.5 * interquartile range. Analysis was performed on all 147,787 gene pairs in the SPIDR library. **f**, UMAP projecting the full set of interactions of each DDR gene with all other DDR genes. Several DDR gene neighborhoods with known, shared functions are highlighted. The UMAP was generated using the strong GEMINI score.

To identify genetic interactions that exceed single-gene effects, we used a variational Bayesian pipeline (GEMINI) that is specialized for the discovery of genetic interactions from CRISPR screening data^14^ (Supplementary Table 3). We found that approximately 18% of the genes in the SPIDR library were individually essential in RPE-1 cells (growth phenotype log_2_ fold change of less than −3). These could not be interrogated for synthetic relationships using the perfectly matched sgRNAs. However, mismatched guide RNAs (Methods) allowed us to blunt individual essential knockdown phenotypes and thereby query their interaction phenotypes (Extended Data Fig. 2a–c). For example, we reidentified interactions between pairs containing essential genes, such as *BRCA2* and *RAD9A*^15^, *CHEK1* and *TOP3A*^16^ (Extended Data Fig. 2c) and Fanconi anaemia pathway components and *RPA1* (ref. ^17^).

We identified approximately 5,000 synthetic lethal interactions (GEMINI score of −1 or less), which corresponds to approximately 3.4% of all queried gene pairs (Extended Data Fig. 2d). Many previously reported genetic interactions were top hits in the SPIDR screen (Fig. 1b). For example, repression of *LIG1* or *POLQ* was synthetic lethal with loss of homologous recombination factors, such as BRCA2 (refs. ^18,19^). *LIG1* also had genetic interactions with both *LIG3* and *XRCC1*, which compensate for LIG1 in Okazaki fragment ligation^20,21^. Genetic interactions identified by SPIDR were systematically enriched for STRING interactions, indicating the ability of our approach to recapitulate known biology (Extended Data Fig. 2e). Clustering SPIDR genetic interaction profiles, we also found that clusters with similar sets of genetic interactions contained physically interacting sub-complexes and genes involved in similar aspects of the DDR tightly grouped together in a uniform manifold approximation and projection (UMAP) embedding of their genetic interaction signatures (Fig. 1c and Extended Data Fig. 2f). These analyses indicated that the SPIDR screen performed well in identifying DDR genetic interactions at massive scale.

In addition to known genetic interactions, we detected many previously unknown interactions with strong GEMINI scores (Fig. 1b). The entire SPIDR dataset is available at https://spidrweb.org, including the ability to interactively query genes of interest and cross-reference to AlphaFold predictions of potential protein complexes^22^.

To explore whether our screen discovered new genetic interactions that might be therapeutically exploited, we mapped tier 1 and 2 cancer genes from the Catalogue of Somatic Mutations in Cancer (COSMIC) and small-molecule targets from the Drug–Gene Interaction Database (DGIdb) onto the DDR network^23,24^. This identified synthetic lethal interactions that connect cancer genes with small-molecule targets (Fig. 1d). For example, *ERCC2* mutations, found in approximately 20% of muscle-invasive bladder cancers, might render these cancers sensitive to DNA-dependent protein kinase catalytic subunit (DNA-PKcs) inhibition^25^.

**Extended Data Fig. 3:**
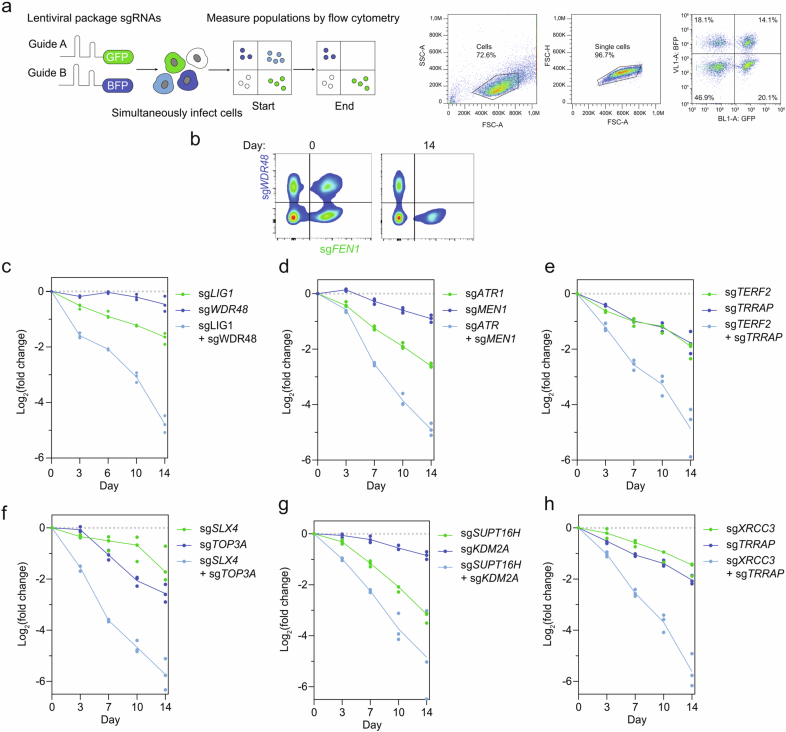
Orthogonal validation of novel synthetic lethal gene pairs. **a**, Left: Schematic representation of the dual-color flow cytometry assay. Cells are transduced with sgRNAs and assayed 96 h later and at regular intervals thereafter. Right: An example of the flow cytometry gating strategy. **b**, Representative flow cytometry density plots of the *FEN1:WDR48* interaction in RPE-1 *TP53* KO dCas9-KRAB cells. **c-h**, RPE-1 *TP53* KO dCas9-KRAB cells were co-transduced with the indicated sgRNAs. Cells populations were first measured by flow cytometry 96 h after transduction (day 0) and on the indicated days thereafter. Log_2_ fold changes relative to day 0 are shown. These panels show the full quantifications of data plotted in Fig. 1e. Source data

We individually tested several of the strongest new genetic interactions using an orthogonal flow cytometry-based method^4^ (Extended Data Fig. 3a,b). We validated the interactions between *LIG1*:*WDR48*, *ATR*:*MEN1*, *TERF2*:*TRRAP*, *SLX4*:*TOP3A*, *XRCC3*:*TRRAP*, *SUPT16H*:*KDM2A*, *FEN1*:*WDR48* and *FANCM*:*SMARCAL1* (Fig. 1e–g and Extended Data Fig. 3b–h). In all cases, the co-depleted cells exhibited a profound and synergistic proliferation defect relative to the corresponding single depletions. These genetic interactions span a wide range of potential molecular functions, from recombination intermediate processing (*SLX4*:*TOP3A*) to epigenome maintenance (*SUPT16H*:*KDM2A*). *TRRAP*, which encodes a member of the TIP60 histone acetyltransferase complex that is recruited to DSBs, has synthetic lethal relationships with the telomere shelterin complex component *TERF2* and the RAD51 paralogue and prostate cancer risk gene *XRCC3* (refs. ^26,27^).

**Extended Data Fig. 4:**
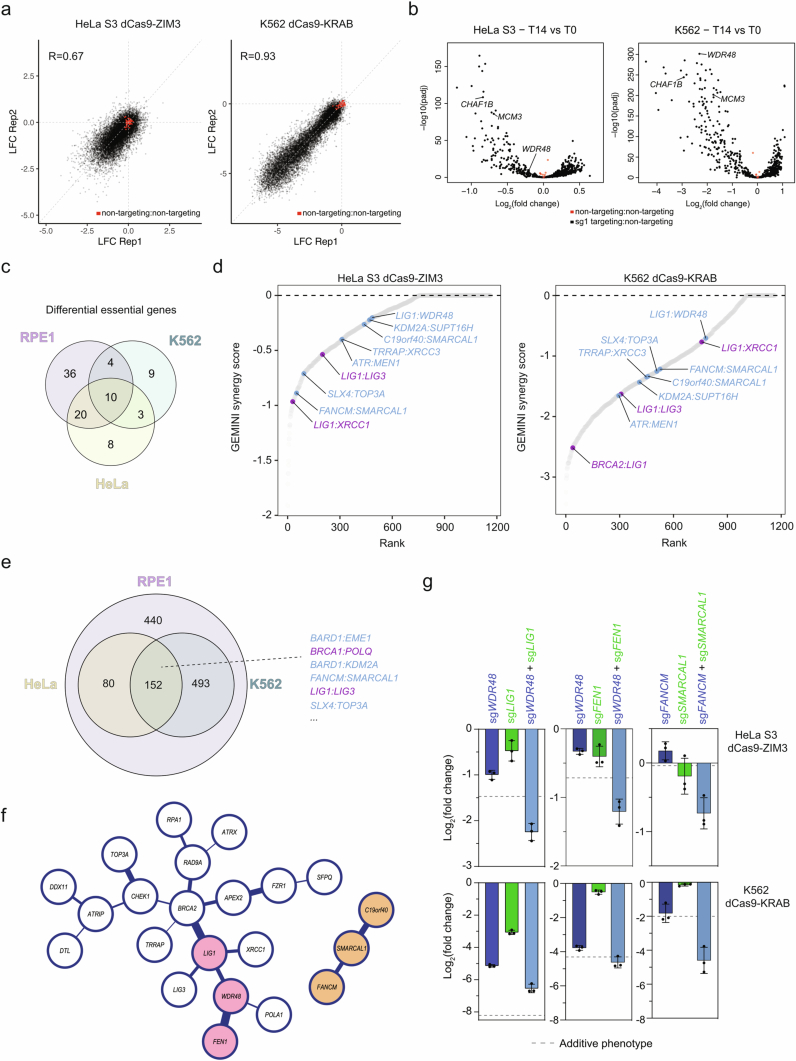
Focused SPIDR screens in HeLa and K562 cells and independent validation of synthetic lethal gene pairs. **a**, LFC comparison T14vsT0 for all library elements. **b**, Volcano plot of log_2_ fold change of non-targeting (non-targeting:non-targeting) and single gene targeting (targeting:non-targeting) library elements at T14 compared to T0. Statistical significance was determined using the Wald test, with Benjamini-Hochberg post-test. **c**, Three-way Venn diagram of essential genes shows differential essential genes identified in the three CRISPRi screens. **d**, Rank order plots of GEMINI sensitive scores in HeLa S3 dCas9-ZIM3 and K562 dCas9-KRAB cells from the focused SPIDR screens. **e**, Three-way Venn diagram showing the intersection of synthetic lethal interactions identified in the three CRISPRi screens. Hits are listed in supplementary table 4. **f**, Stringently filtering for only the strongest synthetic relationships (GEMINI sensitive score ≤−2.5) in RPE-1 cells reveals two unanticipated synthetic lethal modules: *WDR48* (also known as *UAF1*) with *LIG1* and *FEN1*, and *SMARCAL1* with *FANCM* and *C19orf40/FAAP24*. **g**, Dual-color flow cytometry assay performed in HeLa S3 dCas9-ZIM3 and K562 dCas9-KRAB cells. The log_2_ fold changes relative to day 0 are shown. Day 10 values are shown for all pairs except for *FEN1*:*WDR48* (in K562 cells), where day 7 values are shown due to complete dropout of the double transduced population. Error bars represent mean ± s.d. 3 technical replicates are shown. Source data

To identify the synthetic lethal interactions that were consistent in disparate cell lines, we performed dual sgRNA screens targeting the strongest 1,165 gene pairs (GEMINI score −1.5 or less; 14,500 guide-level interactions) in HeLa S3 and K562 cells (Extended Data Fig. 4a). As expected, we found both shared and cell-specific effects for even single-gene perturbations^28^ (Extended Data Fig. 4b,c). Of the queried RPE-1 gene pairs, 62% were also identified in either or both K562 and HeLa S3 cells (Extended Data Fig. 4d,e and Supplementary Table 4). Notably, 13% of RPE-1 synthetic lethal interactions were common to all three cell lines, including *FANCM*:*SMARCAL1*, *SLX4*:*TOP3A* and *LIG1*:*LIG3*. This degree of overlap is in line with dual-guide paralogue screens performed in several cell backgrounds^29^. These data reinforce that our approach captures general as well as cell-specific genetic interactions.

For mechanistic interrogation, we prioritized synthetic lethal gene pairs with (1) the strongest GEMINI scores, (2) one factor that exhibits strong interactions with several members of the same pathway and/or (3) one gene that is frequently mutated in cancer. We focused on two new modules: *WDR48* with *LIG1* and *FEN1*, both Okazaki fragment repair factors, and *SMARCAL1* with *FANCM* and *FAAP24* (also known as *C19orf40*), two genes that encode physically interacting proteins (Fig. 1f,g and Extended Data Fig. 4f). Independent competitive growth assays in HeLa S3 and K562 cells confirmed that these genetic interactions were consistent in at least one other cell line (Extended Data Fig. 4g).

## WDR48–USP1 protect from toxic PCNA ubiquitylation

**Extended Data Fig. 5:**
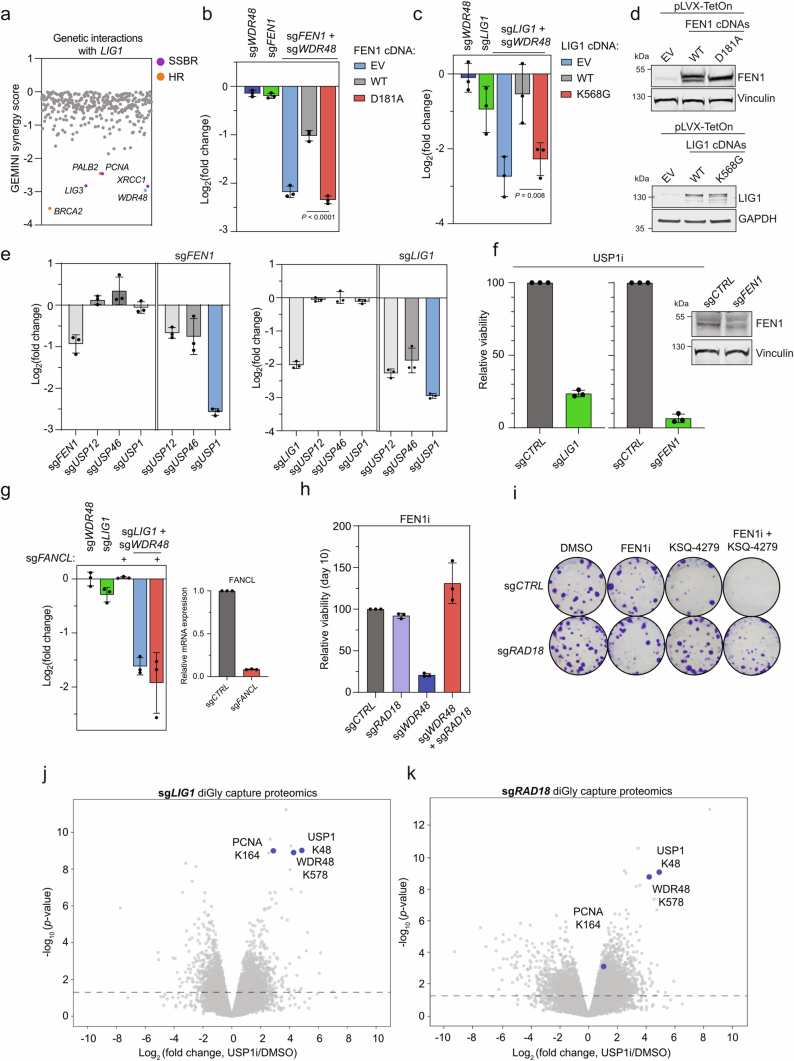
The *LIG1/FEN1:WDR48-USP1* genetic interactions involve the enzymatic activities of LIG1, FEN1, and USP1. **a**, All genetic interactions of *LIG1* identified in the SPIDR screen. **b-c**, Quantification of dual-color competitive growth assays with the indicated (DOX)-inducible FEN1 and LIG1 cDNAs. Dox concentration, 0.5 μg ml^−1^. Day 3 values are shown. Unpaired, two-tailed Student’s *t-*test. **d**, Western blots validating FEN1 and LIG1 cDNA expression (*n* = 1). **e**, Flow cytometric quantifications of the genetic interactions between *FEN1/LIG1* and the genes encoding the WDR48-interacting proteins USP1, USP12, and USP46. Values acquired on day 14 and day 18 are shown respectively. **f**, Quantification of competitive growth assays with cells transduced with the indicated sgRNAs. Cell populations were monitored by flow cytometry after a 12-day treatment with DMSO or USP1i (KSQ-4279, 6 μM). **g**, Left: Dual-color flow cytometry for the *LIG1*:*WDR48* interaction in cells expressing *CTRL* or *FANCL* sgRNA. Right: RT-qPCR data showing relative mRNA expression of FANCL in sg*CTRL* and sg*FANCL* cells. **h**, Quantification of competitive growth assays with cells transduced with the indicated sgRNAs. Cell populations were monitored by flow cytometry after a 10-day treatment with DMSO or FEN1i (10 μM). **i**, Clonogenic survival assay of cells transduced with either a *CTRL* or *RAD18* sgRNA. Cells were cultured for 10 days in the presence of DMSO, FEN1i (5 μM) and/or the USP1 inhibitor KSQ-4279 (2 μM). Data are representative of two experimental replicates. **j-k**, The indicated cells were treated with KSQ-4279 (25 μM, 24 h), and cells lysates were subjected to diGly capture proteomics. The fold change of individual ubiquitylated peptides is shown. Three technical replicates are shown for b, c & e-h. Error bars represent mean ± s.d. Source data

During single-strand DNA (ssDNA) break repair and Okazaki fragment ligation, the FEN1 endonuclease cleaves 5′ DNA flaps and LIG1 seals the resulting ssDNA gap^30,31^. Cells lacking FEN1 or LIG1 accumulate DNA gaps, nicks and/or flaps and rely on both homologous recombination and XRCC1–LIG3 for survival^18,20,21^. In addition to these known interactions, we observed striking new synthetic lethal relationships between both *LIG1* and *FEN1* with *WDR48* (Fig. 1b,e,f and Extended Data Fig. 5a).

In the dual-colour competitive growth assay^4^, expression of wild-type but not catalytically inactive *FEN1* or *LIG1* point mutants rescued cell viability (Extended Data Fig. 5b–d). WDR48 stimulates the activity of the deubiquitylases USP1, USP12 and USP46. Of these, only the knockdown of USP1 was synthetic lethal with FEN1 or LIG1 deficiency (Extended Data Fig. 5e). Moreover, a first-in-class preclinical USP1 inhibitor, KSQ-4279, potently reduced the proliferation of LIG1- or FEN1-depleted cells in competitive growth assays^32^ (Extended Data Fig. 5f). These data indicate that the aberrant accumulation of a ubiquitylated substrate of the WDR48–USP1 complex might underlie its synthetic lethal interactions with *FEN1*/*LIG1*.

**Fig. 2:**
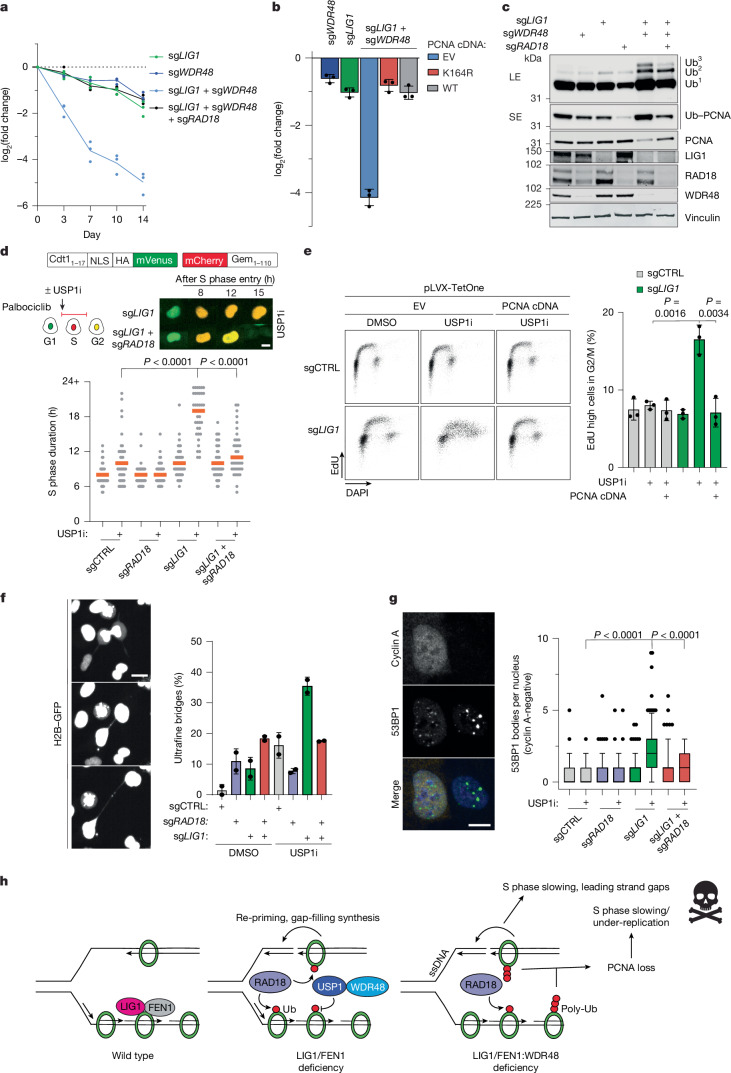
WDR48–USP1 counteracts RAD18-driven PCNA degradation. **a**, Dual-colour flow cytometry assay of the *LIG1*:*WDR48* interaction in cells expressing a control (CTRL) or *RAD18*-targeting sgRNA. Lines represent mean (three technical replicates). **b**, Similar to **a**, but in this case, cells were complemented with the indicated doxycycline (DOX)-inducible cDNAs. Day 10 values are shown. Error bars represent mean ± s.d. (three technical replicates). **c**, Western blot analysis of the indicated proteins. Repeated once with similar results. **d**, Top, schematic of the PIP–FUCCI reporter, experimental set-up and representative images. USP1i, 20 μM. Bottom, quantification of S phase length (two experimental replicates). At least 40 cells were measured per condition. The orange line denotes the median. Statistical analysis was performed using Kruskal–Wallis ANOVA. **e**, Left, representative flow cytometry plots of EdU incorporation and DNA content. Cells were treated with USP1i (25 μM) for 2 days. Samples were treated with 1 μg ml^−1^ DOX. Right, quantification of EdU incorporation in G2/M phase cells (three experimental replicates). Error bars represent mean ± s.d.; unpaired two-tailed Student’s *t*-test. **f**, Left, representative image of a UFB. Right, corresponding quantification (two experimental replicates). Cells stably expressing H2B–GFP were treated with USP1i (25 μM) for 24 h then imaged for an extra 24 h. Error bars represent mean ± s.d.; unpaired two-tailed Student’s *t*-test. **g**, Left, representative images of cyclin A and 53BP1 staining. Cells were treated with USP1i (25 μM) for 48 h. Right, corresponding quantification. Box plots show median values and data within the 10–90 percentile. The lower and upper ends indicate the 25th and 75th percentiles. At least 75 cells from two experimental replicates were quantified. Statistical analysis was performed using Kruskal–Wallis ANOVA. **h**, A model for the *FEN1*/*LIG1*:*WDR48–USP1* synthetic lethal interactions. In **d**–**g**, USP1i refers to KSQ-4279. EV, empty vector; LE, long exposure; SE, short exposure; Ub, ubiquitylated; WT, wild type. Scale bars, 10 μm (**d**,**g**), 25 μm (**f**). Source data

WDR48–USP1 counteracts ubiquitylation by the FANCL and RAD18 ubiquitin ligases^33,34^. In dual-colour competitive growth assays, depletion of RAD18 but not FANCL completely rescued the growth defect of LIG1:WDR48-deficient cells (Fig. 2a and Extended Data Fig. 5g). Similarly, co-depletion of RAD18 completely reversed the high sensitivity of WDR48-depleted cells to FEN1 inhibitor (FEN1i) treatment (Extended Data Fig. 5h). Clonogenic survival assays confirmed that FEN1i and KSQ-4279 co-treatment is synthetic lethal in a RAD18-dependent manner (Extended Data Fig. 5i).

To identify RAD18 substrates that might drive *LIG1/FEN1*:*WDR48–USP1* synthetic lethality, we performed unbiased diGly capture proteomics. This identified a more than sevenfold increase in ubiquitylation of lysine (K)164 on PCNA upon USP1 inhibition in LIG1-depleted cells (Extended Data Fig. 5j,k). Comparing the effects of USP1 inhibition between LIG1- and RAD18-depleted cells, there was approximately 3.5-fold less PCNA K164 ubiquitylation in RAD18-depleted cells than LIG1-depleted cells after USP1 inhibition. These data pinpointed PCNA as a potential substrate of RAD18 that drives cell death in LIG1/FEN1-deficient cells if hyper-ubiquitylation cannot be reversed by WDR48-USP1.

**Extended Data Fig. 6:**
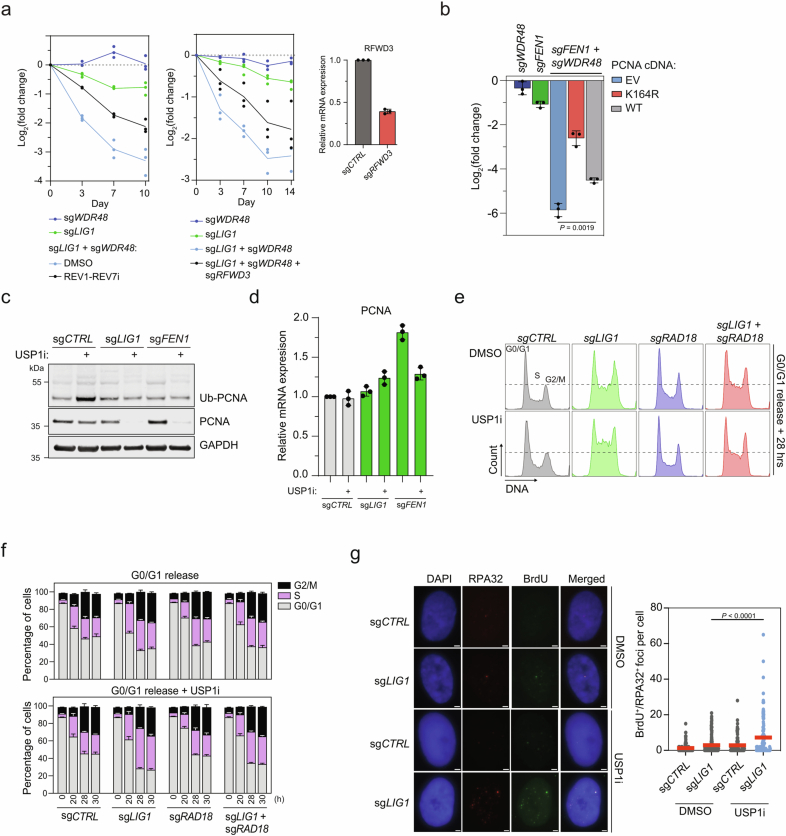
The *LIG1/FEN1:WDR48-USP1* genetic interactions are driven by low PCNA protein levels and associated with replication gaps and a longer S phase. **a**, Left: Dual-color flow cytometry assay of the *LIG1*:*WDR48* interaction in cells that were treated with the REV1-REV7 interface inhibitor, JH-RE-06 (REV1-REV7i, 1 μM) or expressing *CTRL* or *RFWD3* sgRNA. Right: RT-qPCR data showing relative mRNA expression of RFWD3 in sg*CTRL* and sg*RFWD3* cells. **b**, Dual-color flow cytometry assay of FEN1, WDR48, and co-depleted cells complemented with the indicated doxycycline (DOX)-inducible cDNAs. Dox concentration 1 μg ml^−1^ (for all PCNA cDNAs). Day 10 values are shown. Unpaired, two-tailed Student’s *t-*test. **c**, Western blot showing PCNA and ubiquitylated PCNA levels in sg*CTRL*, sg*LIG1* or sg*FEN1* cells after a 7-day treatment with DMSO or USP1i (KSQ-4279, 25 μM). **d**, RT-qPCR data of relative *PCNA* mRNA expression in the cells in (a). **e**, Representative flow cytometry profiles of DNA content (DAPI) analysis in cells transduced with the indicated sgRNAs 28 hrs after release from G0/G1. G0/G1 arrested cells were released into the cell cycle in the presence of DMSO or the USP1 inhibitor ML-323 (USP1i; 30 μM). **f**, Quantification of (e) with all timepoints. **g**, Representative images and quantification of BrdU/RPA32 staining of sg*CTRL* or sg*LIG1* cells after a 7-day treatment with DMSO or USP1i (KSQ-4279, 25 μM). Red line denotes the mean; unpaired, two-tailed Student’s *t-*test. A minimum of 100 cells derived from two experimental replicates were measured per condition. Scale bar, 2 μm. 3 technical replicates are shown for a, b & d-f. Error bars represent mean ± s.d. Source data

RAD18-mediated PCNA ubiquitylation could cause cell death by triggering either overactive translesion synthesis (TLS) or degradation of PCNA^35^. We ruled out overactive TLS in two ways: first, we used the small-molecule JH-RE-06 to prevent the interaction of REV1 with the REV7 subunit of DNA polymerase ζ, the major TLS extension polymerase; second, we depleted the E3 ubiquitin ligase RFWD3, which ubiquitylates PCNA downstream of RAD18 to promote TLS polymerase recruitment to PCNA^36^. Both interventions temporarily delayed but did not prevent the *LIG1*:*WDR48* synthetic lethality (Extended Data Fig. 6a). Hence, overactive TLS was not the ultimate cause of cell death.

We tested whether degradation-driven paucity of PCNA drove the synthetic lethality in four ways. First, we overexpressed wild-type PCNA or PCNA mutated at the RAD18 ubiquitylation site, which we found through unbiased diGly proteomics (K164R). Both were sufficient to completely rescue the growth defect of LIG1:WDR48 co-depleted cells and partially rescue FEN1:WDR48 co-deficient cells (Fig. 2b and Extended Data Fig. 6b). Second, we found that the levels of non-ubiquitylated PCNA were substantially reduced in cells co-depleted of LIG1 and WDR48 (Fig. 2c) and in LIG1- or FEN1-depleted cells treated with a USP1 inhibitor (USP1i; Extended Data Fig. 6c). Third, PCNA transcript levels were not significantly reduced in LIG1- or FEN1-depleted cells treated with USP1i, consistent with post-transcriptional regulation (Extended Data Fig. 6d). Fourth, RAD18 depletion blunted PCNA ubiquitylation and reversed its loss in LIG1-depleted cells (Fig. 2c), suggesting that PCNA mono-ubiquitylation ultimately leads to its poly-ubiquitylation and degradation if not counteracted by WDR48–USP1. Overall, our data support a model in which RAD18-dependent PCNA degradation is the major driver of *LIG1*/*FEN1*:*WDR48* synthetic lethality.

Lack of PCNA should disrupt DNA replication and cell cycle progression. To test this, we synchronized cells in the G0/G1 phase and monitored their progression through the cell cycle. Consistent with a slower S phase, LIG1-depleted cells treated with USP1i entered the S phase normally but accumulated in the S phase at later time points. RAD18 depletion rescued this phenotype, with corresponding increases in G1 and G2/M phase cells (Extended Data Fig. 6e,f). To directly monitor the length of the S phase, we performed live imaging of cells expressing a PIP–FUCCI reporter that were arrested in the G1 phase and then released in the presence of USP1i^37^. LIG1-depleted cells treated with USP1i exhibited a pronounced RAD18-dependent S phase lengthening (Fig. 2d).

**Extended Data Fig. 7:**
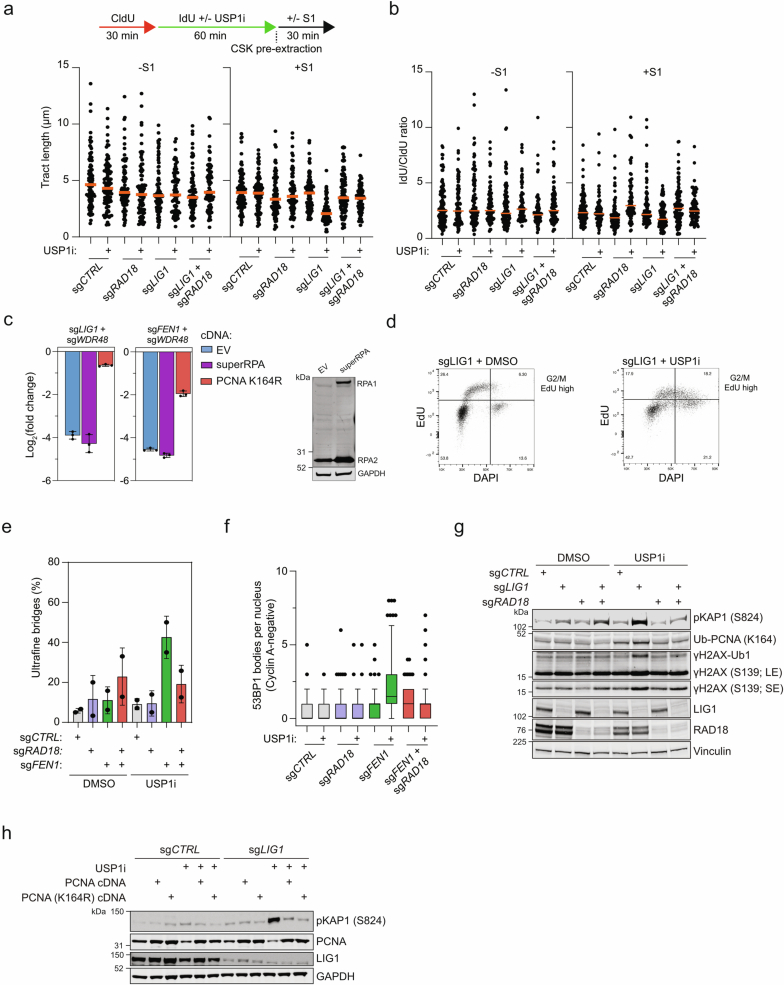
Low PCNA protein levels lead to ssDNA gaps, a longer S phase, ultrafine bridges, under-replicated DNA, and DNA damage. **a**, Top: schematic of the S1 nuclease DNA fiber assay. Where indicated, cells were treated with KSQ-4279 (25 μM). Bottom: measurements of IdU tract lengths in the indicated cells, with and without S1 nuclease treatment. A minimum of 70 tracts from one experiment were measured per condition. Orange line denotes the median. **b**, Measurements of IdU/CIdU ratio for fibers shown in Extended Data Fig. 7a. A minimum of 70 tracts from one experiment were measured per condition. Orange line denotes the median. **c**, Left: Quantification of the dual-color flow cytometry assay with indicated cDNAs. 3 technical replicates are shown. Error bars represent mean ± s.d. Right: Western blot of indicated cells. Expression was induced with 1 µg ml^−1^ doxycycline. **d**, Representative gating of EdU-high cells in G2/M phases. Corresponds to data shown in Fig. 2e. **e**, Quantification of ultrafine anaphase bridges for sg*FEN1* cells. Data are representative of two experimental replicates. Error bars represent mean ± s.d. **f**, Quantification of 53BP1 nuclear bodies for sg*FEN1* cells. Whisker box plots show median values and data within the 10–90 percentile. The lower and upper ends of the boxplot indicate the 25th and 75th percentile values. At least 75 cells were quantified from two experimental replicates. The values for sg*CTRL* and sg*RAD18* samples are the same as those in Fig. 2g. **g**, Western blot of cells transduced with the indicated sgRNAs and treated with DMSO or the USP1 inhibitor (USP1i) ML-323 (30 μM) for 7 days. LE, long exposure; SE, short exposure. Phosphorylated (p)KAP1 on serine (S) 824 and γH2AX ubiquitylation (Ub) serve as markers of DSB signalling. **h**, Western blot of cells transduced with the indicated sgRNAs and cDNAs, treated with DMSO or the USP1i, KSQ-4279 (25 μM) for 7 days. g-h were each performed once. Source data

We explored whether the slowed cell cycle progression caused by PCNA ubiquitylation and degradation was associated with impaired DNA replication. Indeed, USP1 inhibition exacerbated the accumulation of stretches of RPA-bound ssDNA in LIG1-depleted cells, which potentially arise from replication gaps (Extended Data Fig. 6g). To explicitly test for the presence of gaps, we used the DNA fibre assay. When permeabilized cells were treated with S1 nuclease, which cleaves ssDNA gaps, the replication tracts of USP1-inhibited LIG1-depleted cells were further shortened to a greater extent than in control cells (Extended Data Fig. 7a,b). RAD18 depletion reversed this shortening, confirming that WDR48–USP1 is required to limit post-replicative ssDNA gap accumulation in LIG1-depleted cells.

Accumulation of ssDNA could lead to RPA exhaustion and cell death^38^. However, overexpression of all RPA subunits failed to rescue the viability of cells (Extended Data Fig. 7c).

An alternative hypothesis is that PCNA degradation and perturbed DNA replication in LIG1/FEN1:WDR48–USP1-deficient cells causes cells to enter mitosis with under-replicated DNA. We observed that a higher proportion of LIG1-deficient cells treated with USP1i incorporated 5-ethynyl-2′-deoxyuridine (EdU) in the G2 phase of the cell cycle, reflecting an effort to salvage under-replicated DNA before cell division^39^ (Fig. 2e and Extended Data Fig. 7d). PCNA overexpression completely rescued this aberrant EdU incorporation profile, consistent with PCNA being a limiting factor in LIG1 and USP1 co-depleted cells. USP1 inhibition also exacerbated ultrafine bridge (UFB) formation, a hallmark of DNA under-replication, in LIG1- or FEN1-depleted cells, and this was alleviated by RAD18 co-depletion (Fig. 2f and Extended Data Fig. 7e). If unresolved in mitosis, UFBs are prone to breakage, leading to the formation of 53BP1 bodies in the subsequent G1 phase^40^. Indeed, there was a pronounced RAD18-driven accumulation of 53BP1 bodies in LIG1- or FEN1-depleted cells treated with USP1i (Fig. 2g and Extended Data Fig. 7f). RAD18 depletion or PCNA overexpression suppressed this DSB signalling in USP1-inhibited LIG1-depleted cells (Extended Data Fig. 7g,h). Thus, USP1 inhibition causes severe DNA under-replication and genomic instability in both LIG1- and FEN1-depleted cells.

Overall, our data support a model in which WDR48–USP1 counteract the hyperactive RAD18-mediated ubiquitylation of PCNA in FEN1/LIG1-depleted cells (Fig. 2h). In cells depleted of FEN1 or LIG1, accumulating DNA gaps trigger PCNA ubiquitylation. Without balancing the WDR48–USP1 activity, this accumulates to hyper-ubiquitylation and PCNA degradation. The lack of PCNA ultimately leads to severe DNA under-replication and genomic instability. This function of WDR48–USP1 in preventing PCNA degradation may also be important for the subset of Okazaki fragments that evade repair by FEN1 and LIG1 (ref. ^20^). Our data further imply a use for USP1 inhibitors in tumours where ssDNA gaps accumulate, such as *FEN1*-mutant colorectal cancers^41^.

## FANCM and SMARCAL1 prevent breaks at TA repeats

We next turned to the interactions of *FANCM* and *FAAP24* with *SMARCAL1* as a second set of strong and new genetic interactions identified in the SPIDR screen (Fig. 1b,g and Extended Data Fig. 4f). FAAP24 and FANCM are physical interaction partners with roles in interstrand crosslink (ICL) repair and replication fork stalling^42,43^. FANCM is recruited to substrates by FAAP24, possesses ATP-dependent translocase activity and acts as a scaffold for numerous other DNA repair proteins^44^. The SMARCAL1 translocase facilitates the remodelling of stalled DNA replication forks alongside ZRANB3 and HLTF^45,46^. Individual depletion of SMARCAL1, ZRANB3 or HLTF suppresses replication fork reversal, and these enzymes are thought to cooperatively regulate this process^47^.

**Fig. 3:**
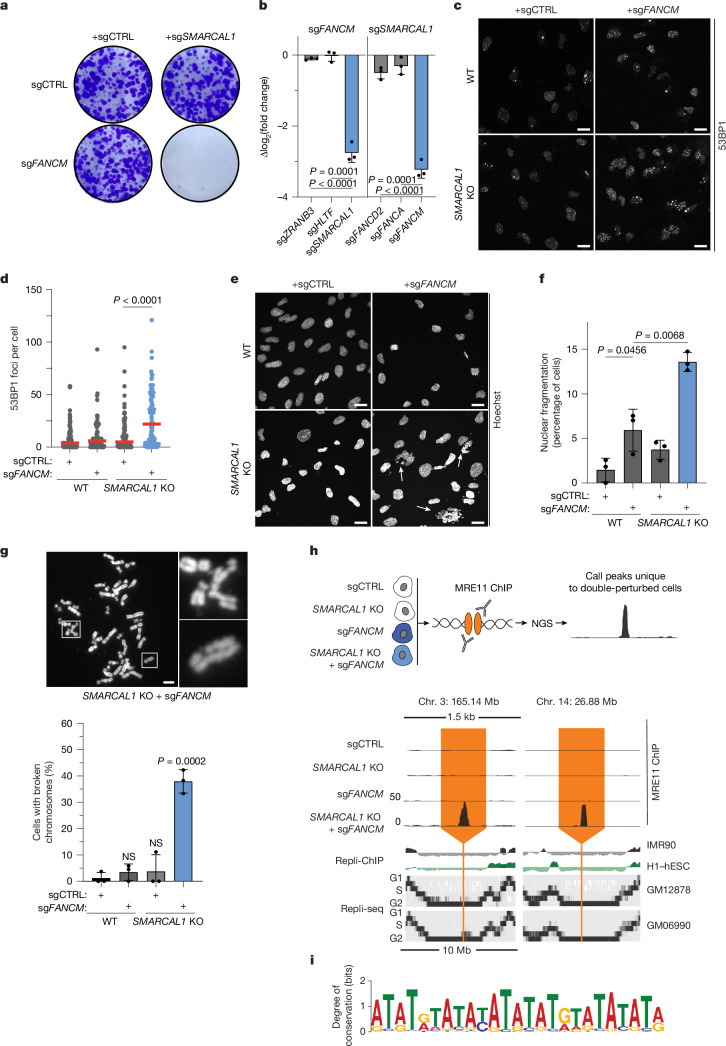
Combined FANCM and SMARCAL1 loss leads to DSBs at late-replicating TA-rich repeats. **a**, Co-depletion of FANCM and SMARCAL1 reduces clonogenic survival. Cells were transduced with sgRNA-containing lentiviruses, co-expressed with either puromycin or neomycin resistance cassettes. Representative images of 10-day clonogenic survival are shown. Data are representative of two experimental replicates. **b**, Competitive growth assays for cells co-transduced with the indicated sgRNAs. The log_2_ fold change of co-depleted cells in a flow cytometry assay was normalized to the corresponding single depletions (sg*FANCM* or sg*SMARCAL1*). Values acquired on day 14 are shown. Error bars represent mean ± s.d.; unpaired two-tailed Student’s *t*-test. Three technical replicates from one experiment are shown. **c**, Representative images of 53BP1 foci in either wild-type or *SMARCAL1* KO cells 7 days post-transduction with a non-targeting or *FANCM*-targeting sgRNA. **d**, Quantification of 53BP1 focus formation. Red line denotes the median; two-tailed Mann–Whitney test. A minimum of 80 cells from two experimental replicates were measured per condition. **e**, Representative images of nuclei, stained with Hoechst, in the indicated cells. White arrows indicate cells exhibiting nuclear fragmentation. **f**, Quantification of nuclear fragmentation events from three experimental replicates. Error bars represent mean ± s.d.; unpaired two-tailed Student’s *t*-test. **g**, Top, a representative metaphase spread from *SMARCAL1* KO cells co-depleted of *FANCM*. Bottom, quantification of metaphases with at least one broken chromosome from three independent experiments. Error bars represent mean ± s.d.; unpaired two-tailed Student’s *t*-test. **h**, Top, a schematic of the MRE11 ChIP–seq approach. Three independent ChIP–seq experiments were performed. Bottom, representative MRE11 ChIP–seq tracks spanning a 1.5-kb window aligned with Repli-ChIP and Repli-seq tracks spanning a 10-Mb window in IMR90, H1-hESC, GM12878 and GM06990 cells. **i**, Consensus motif identified by performing Multiple Expectation maximizations for Motif Elicitation (MEME) analysis on DNA sequences underlying 72 MRE11 ChIP–seq peaks found only in *SMARCAL1* KO:sg*FANCM* cells. NGS, next-generation sequencing; NS, not significant (*P* > 0.05). Scale bars, 20 μm (**c**,**e**), 10 μm (**g**). Source data

**Extended Data Fig. 8:**
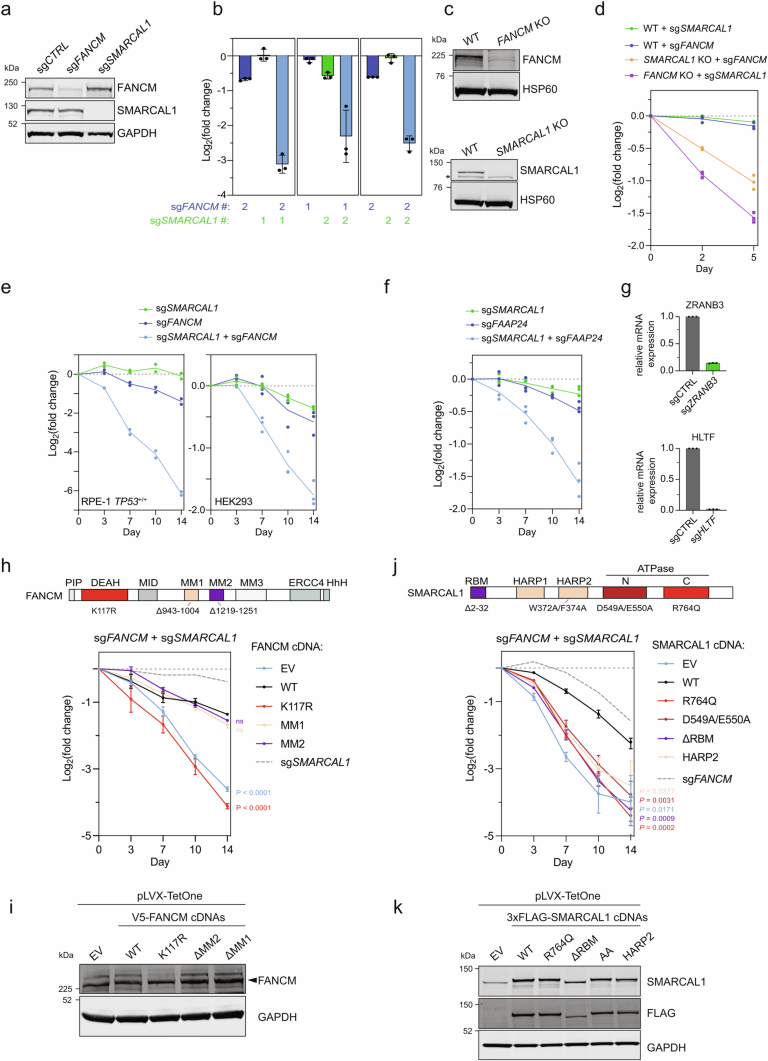
Validation and characterization of the *FANCM*:*SMARCAL1* synthetic lethal interaction. **a**, Western blot validating sgRNA-mediated knockdown of *FANCM* and *SMARCAL1* (*n* = 1). **b**, Quantifications of the dual-color flow cytometry assay measurements of cells transduced with the indicated sgRNAs. The log_2_ fold change on day 14 is shown. Error bars represent mean ± s.d. of 3 technical replicates. **c**, Western blot of *FANCM* and *SMARCAL1* knockout (KO) cells (*n* = 1). The asterisk denotes a non-specific band. **d**, Competitive growth assays of wild-type (WT) and *FANCM* or *SMARCAL1* KO RPE-1 *TP53* KO dCas9-KRAB cells transduced with the indicated sgRNAs with the same MOI. Values were normalized to day 0. **e**, Dual-color flow cytometry assay of dCas9-KRAB-expressing RPE-1 *TP53*-proficient and HEK293 cells transduced with the indicated sgRNAs. Log_2_ fold changes relative to day 0 are shown. **f**, Quantification of the dual-color assay for cells expressing sgRNAs targeting *FAAP24* and/or *SMARCAL1*. **g**, Validation of CRISPRi-mediated knockdown of *ZRANB3* and *HLTF* by RT-qPCR. mRNA expression was normalized to the sg*CTRL* sample. 3 technical replicates are shown. **h**, Quantification of the dual-color flow cytometry assay for FANCM:SMARCAL1 co-depleted cells complemented with the indicated *FANCM* cDNAs. cDNA expression was induced with 1 µg ml^−1^ doxycycline. The log_2_ fold change was normalized to Day 0. Error bars represent mean ± s.d. **i**, Western blot controls of cDNA expression for Extended Data Fig. 8h. **j**, Dual-color flow cytometry assay for FANCM:SMARCAL1 co-depleted cells complemented with the indicated *SMARCAL1* cDNAs. Error bars represent mean ± s.d. Experiments were performed as in Extended Data Fig. 8h, except cDNA expression was induced with 0.25 µg/ml^−1^ doxycycline. **k**, Western blot controls of cDNA expression for Extended Data Fig. 8j. Unpaired, two-tailed Student’s *t-*tests compare cells expressing WT cDNAs with each other condition on day 14; ns = not significant (*P* > 0.05); Three technical replicates are shown for b, d, e, f, h, and j. Source data

Co-depletion of FANCM and SMARCAL1 profoundly reduced clonogenic survival (Fig. 3a and Extended Data Fig. 8a). The combined loss of FANCM and SMARCAL1 was synthetic lethal using two different sgRNAs targeting each gene and upon knockdown of either FANCM or SMARCAL1 in clonal cell lines where the other partner was knocked out (Extended Data Fig. 8b–d). The genetic interaction was consistent in HEK293, HeLa S3, K562 and p53-proficient RPE-1 cells, demonstrating that it is not cell line specific and is independent of p53 status (Extended Data Figs. 4g and 8e). Co-depletion of FAAP24 and SMARCAL1 was also synthetic lethal in individual assays (Extended Data Fig. 8f).

FANCM and SMARCAL1 are part of multicomponent pathways; therefore, we examined whether each gene had synthetic lethal relationships with other components of these respective pathways. We observed no genetic interaction between *SMARCAL1* and Fanconi anaemia genes involved in ICL repair and homologous recombination, such as *FANCD2* or *FANCA* (Fig. 3b). FANCM deficiency was not synthetic lethal with loss of the other replication fork reversal enzymes ZRANB3 and HLTF (Fig. 3b and Extended Data Fig. 8g). The specificity of the *FANCM*:*SMARCAL1* genetic interaction implies a new and potentially shared function for each factor.

To delineate which domains of FANCM and SMARCAL1 are involved in their genetic interaction, we performed dual-colour flow cytometry assays in cells expressing complementary DNAs (cDNAs) with point mutations or deletions (Extended Data Fig. 8h–k). Wild-type *FANCM* and variants lacking the Fanconi anaemia core complex-binding MM1 (Δ943–1004) or BLM–TOP3A–RMI complex-binding MM2 domain (Δ1,219–1,251) rescued the synthetic lethality. However, an ATPase-defective mutant (K117R) did not (Extended Data Fig. 8h,i). This indicates that the DNA-remodelling motor function of FANCM is required in SMARCAL1-deficient cells, but its recruitment of the Fanconi anaemia and BLM–TOP3A–RMI complexes is dispensable.

Reciprocally, we tested whether *SMARCAL1* variants lacking the N-terminal RPA-binding motif (RBM) (Δ2–32), the HARP2 domain required for DNA binding/annealing (W372A/F374A) or ATPase activity (R764Q and D549A/E550A) affected the synthetic lethal phenotype (Extended Data Fig. 8j,k). Expression of the ΔRBM, HARP2 and both ATPase-dead variants failed to rescue the viability, unlike wild-type *SMARCAL1*. This suggests that the ability of SMARCAL1 to bind both RPA and DNA and its motor-driven annealing activity are required for cell survival in the absence of FANCM.

**Extended Data Fig. 9:**
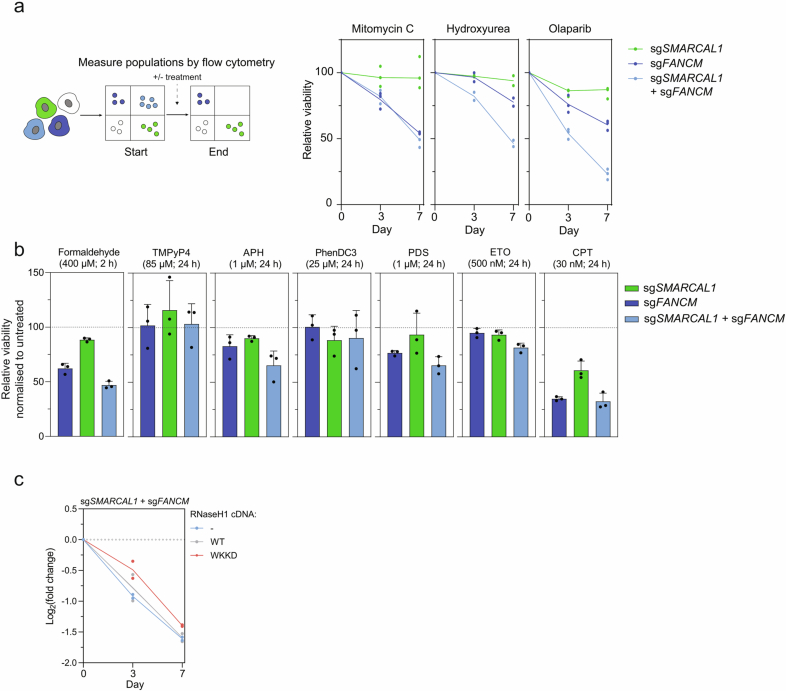
Related to Fig. 3. *FANCM:SMARCAL1*-deficient cells are highly sensitive to hydroxyurea or Olaparib. **a**, Left: schematic representation of the drug sensitivity assay. Right: dual-color flow cytometry survival curves for cells expressing the indicated sgRNAs and treated with mitomycin C (5 nM, 24 h; *n* = 3 technical replicates), hydroxyurea (2 mM, 24 h; *n* = 2 experimental replicates), or Olaparib (2 µM, chronic; *n* = 3 technical replicates). Cell population ratios were normalized to those of corresponding untreated cells. A full stress sensitization panel can be found in (b). **b**, Cells were treated with genotoxic agents (APH, aphidicolin; PDS, pyridostatin; ETO, etoposide; CPT, camptothecin) 96 hrs after transduction with the indicated sgRNAs. Concentrations and treatment durations are indicated above each panel. Cells were re-analysed by FACS 7 days later. Cell population ratios were normalized to those of corresponding untreated cells. Three technical replicates are shown. Error bars represent mean ± s.d. **c**, Survival curves of RPE-1 *TP53* KO cells transduced with sgRNAs targeting *FANCM* and *SMARCAL1* and nucleofected with a cDNA encoding either wild-type (WT) or R-loop-binding defective (WKKD) RNase H1. Source data

Our data pointed to a new function of SMARCAL1 that is independent of the fork remodelling activity shared with ZRANB3 and HLTF. We used exogenous DNA damaging agents to identify what type of lesion was driving the *FANCM*:*SMARCAL1* synthetic lethality. We found that co-depleted cells were not further sensitized to ICLs, G4 quadruplex stabilization, replication fork stalling at common fragile sites or topoisomerase trapping (Extended Data Fig. 9a,b). Overexpression of RNase H1 failed to rescue the synthetic lethal phenotype, indicating that RNA–DNA hybrids are not involved (Extended Data Fig. 9c). However, FANCM:SMARCAL1 co-depleted cells exhibited hypersensitivity to hydroxyurea and the PARP inhibitor, olaparib, which induce DNA replication stress through distinct mechanisms (Extended Data Fig. 9a).

Replication stress can lead to DSB formation. Indeed, even in the absence of exogenous DNA damaging agents, *SMARCAL1* KO cells co-depleted of FANCM contained many more DSBs as quantified by 53BP1 foci (Fig. 3c,d). These cells also displayed high levels of nuclear fragmentation, a hallmark of mitotic DNA damage^38^ (Fig. 3e,f). Chromosome spreads revealed that double-perturbed cells exhibited elevated frequencies of broken chromosomes in metaphase (Fig. 3g). These data suggest that simultaneous loss of FANCM and SMARCAL1 results in DSBs that persist into mitosis.

**Extended Data Fig. 10:**
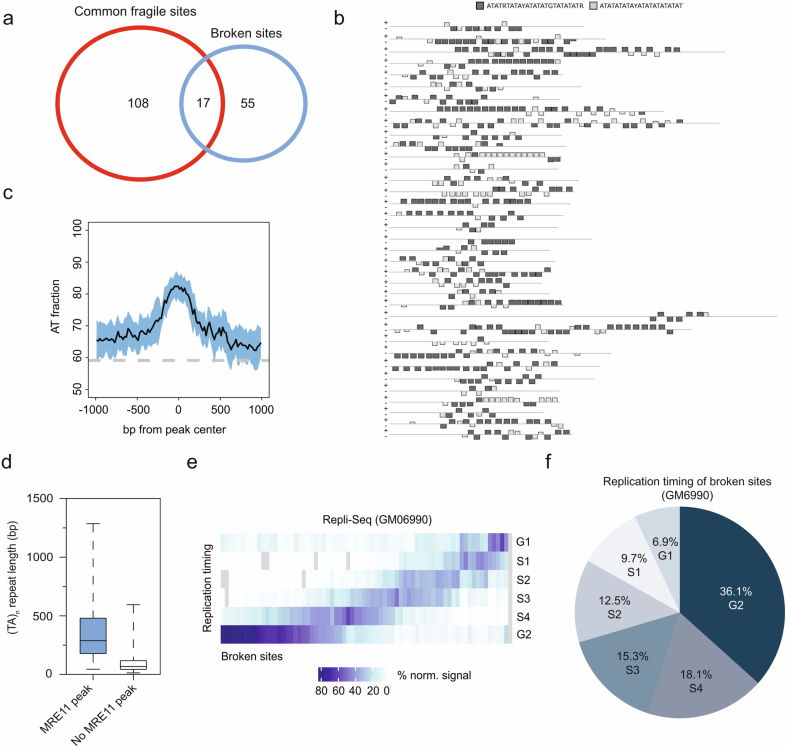
Related to Fig. 3. Further characterization of late-replicating TA-rich DSB sites. **a**, A Venn diagram showing the intersection between the 72 break sites and the 125 common fragile sites in the humCFS database. **b**, Example screenshot of MAST output from the 72 break sites. The most common motifs are shown in dark and light gray. Peak sequences contain multiple copies of the respective motifs. **c**, Plot showing the AT fraction per 20 base pair adjacent windows across 72 MRE11 ChIP-Seq peaks only found in the *SMARCAL1* KO:sg*FANCM* cells. Peaks were aligned according to their peak center. The AT fraction was calculated per 20 base pair in a 1 kb window. Blackline indicates the mean and blue area indicates standard deviation. The average AT fraction of the human genome is shown as a grey dashed line. **d**, Boxplot showing the lengths (1-99 percentile) of all annotated TA repeats in the hg19 reference genome, grouped according to whether or not they overlap an MRE11 ChIP-Seq peak exclusively detected in *SMARCAL1* KO:sg*FANCM* cells. The lower and upper ends of the boxplot indicate the 25th and 75th percentile values. Centre line represents median. 69/72 MRE11 peaks overlapped an annotated TA repeat. 66,575 annotated TA repeats do not overlap MRE11 peaks. The locations and lengths of (TA)_n_ repeats in the hg19 reference genome were taken from van Wietmarschen et al., 2020. **e**, Heatmap showing percentage-normalized signal from Repli-seq data in GM06990 cells across 72 MRE11 ChIP-Seq peaks only found in the *SMARCAL1* KO:sg*FANCM* cells. The average signal for each phase was taken per peak and ordered from late to early replication timing. **f** Pie chart showing 72 MRE11 ChIP-Seq peaks only found in the *SMARCAL1* KO:sg*FANCM* cells categorized into replication timing phases based on the maximum signal below each peak using percentage-normalized signal tracks from Repli-seq data in GM06990 cells. Source data

To determine the genomic context in which FANCM and SMARCAL1 are required to ensure chromosomal stability, we performed MRE11 chromatin immunoprecipitation sequencing (ChIP–seq)^48,49^. This approach identified recurrent sites of DSBs in wild-type, *SMARCAL1* KO, FANCM-depleted and double-perturbed cells^48,49^ (Fig. 3h and Supplementary Table 5). Relative to wild-type and single perturbation, double-perturbed cells exhibited very strong MRE11 binding, indicating DSBs, at 72 unique genomic loci. Many of these overlapped known common fragile sites^50^ (Extended Data Fig. 10a). Motif analysis revealed a strong enrichment of interrupted TA-rich DNA sequences at these breakpoints (Fig. 3i and Extended Data Fig. 10b,c), and MRE11-bound TA repeats were much longer than unbound TA repeats (Extended Data Fig. 10d). Strikingly, the breaks in *SMARCAL1* KO:sg*FANCM* cells were largely present in very late replicating regions^51^ (Fig. 3h and Extended Data Fig. 10e,f). Late formation of these DSBs potentially makes them less likely to be detected and could explain why they are not repaired before cells enter mitosis.

**Fig. 4:**
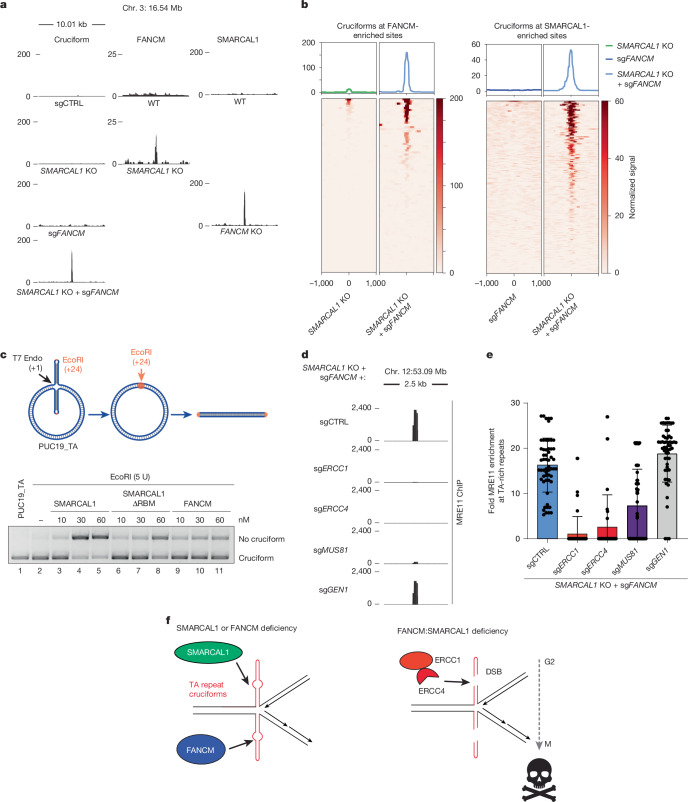
FANCM:SMARCAL1-deficient cells accumulate cruciforms that are predominantly cleaved by ERCC1–ERCC4. **a**, Representative cruciform, FANCM and SMARCAL1 ChIP–seq (*n* = 1) tracks spanning a 10-kb window on chromosome 3. **b**, Profile plots and heat maps showing the intensity of cruciform signal at sites where FANCM is enriched in *SMARCAL1* KO cells and sites where SMARCAL1 is enriched in *FANCM* KO cells. **c**, Top, schematic representation of the cruciform unfolding assay on a model TA repeat substrate. Bottom, unfolding of TA-cruciform DNA by SMARCAL1, SMARCAL1 ΔRBM and FANCM (data are representative of three experimental replicates). The presence of the cruciform secondary structure was estimated by DNA cutting with EcoRI, which does not cut hairpins at cruciform DNA but cuts double-stranded DNA. **d**, Representative MRE11 ChIP–seq (*n* = 1) tracks from *SMARCAL1* KO:sg*FANCM* cells that also have knockdown of one of the indicated nucleases. **e**, Quantification of MRE11 enrichment at 35 sites in FANCM:SMARCAL1-deficient cells that also have knockdown of one of the indicated nucleases (*n* = 1; error bars represent mean ± s.d.). **f**, A model depicting the compensating activities of FANCM and SMARCAL1 in maintaining genome stability at TA repeats. Complementary action of FANCM and SMARCAL1 directly unfolds cruciforms that form at TA-rich repeats. When both SMARCAL1 and FANCM are absent, cruciforms are cleaved by ERCC1–ERCC4, leading to DSB formation upon entry into mitosis. Source data

**Extended Data Fig. 11:**
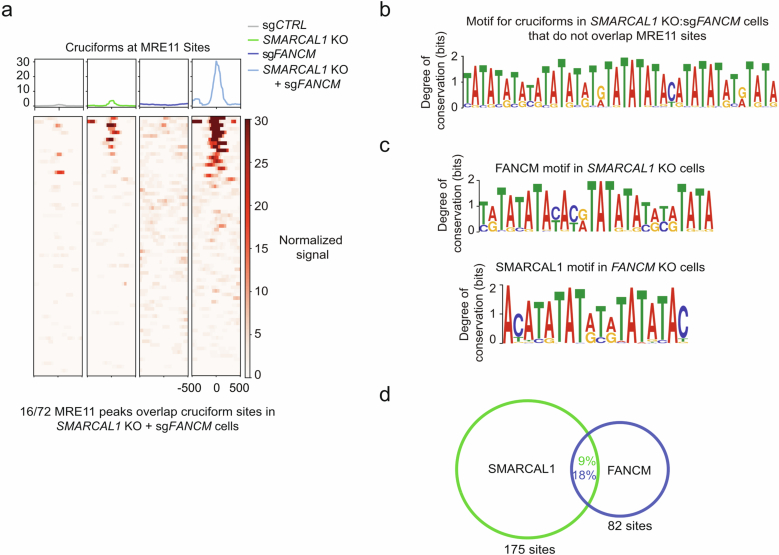
Related to Fig. 4. Cruciform formation at MRE11 sites and characterisation of FANCM and SMARCAL1 binding sites. **a**, Profile plots and heat maps showing the intensity of cruciform signal at sites where MRE11 is enriched in the indicated cells. **b**, Consensus motif identified by performing MEME analysis on DNA sequences underlying cruciform peaks identified in *SMARCAL1* KO:sg*FANCM* cells that do not overlap MRE11 sites. **c**, MEME analysis on DNA sequences underlying FANCM ChIP-Seq peaks in *SMARCAL1* KO cells and SMARCAL1 peaks in *FANCM* KO cells. **d**, Venn diagram showing the overlap between ChIP-Seq peaks for SMARCAL1 and FANCM.

TA repeats are prone to form secondary structures, such as cruciforms^7,52^. Using ChIP–seq^53^, we detected significant enrichment of cruciforms at TA repeats in *SMARCAL1* KO:sg*FANCM* cells, but not in cells individually depleted of either factor (Fig. 4a, Extended Data Fig. 11a,b and Supplementary Table 5). To directly test if FANCM and SMARCAL1 are recruited to cruciforms forming at TA repeats, we determined the genome-wide binding of each translocase by ChIP–seq. We observed enrichment of FANCM at TA repeats only in *SMARCAL1* KO cells and enrichment of SMARCAL1 at TA repeats only in *FANCM* KO cells (Fig. 4a, Extended Data Fig. 11c,d and Supplementary Table 5). Binding of each translocase overlapped the cruciform sites in double-perturbed cells (Fig. 4a,b).

**Extended Data Fig. 12:**
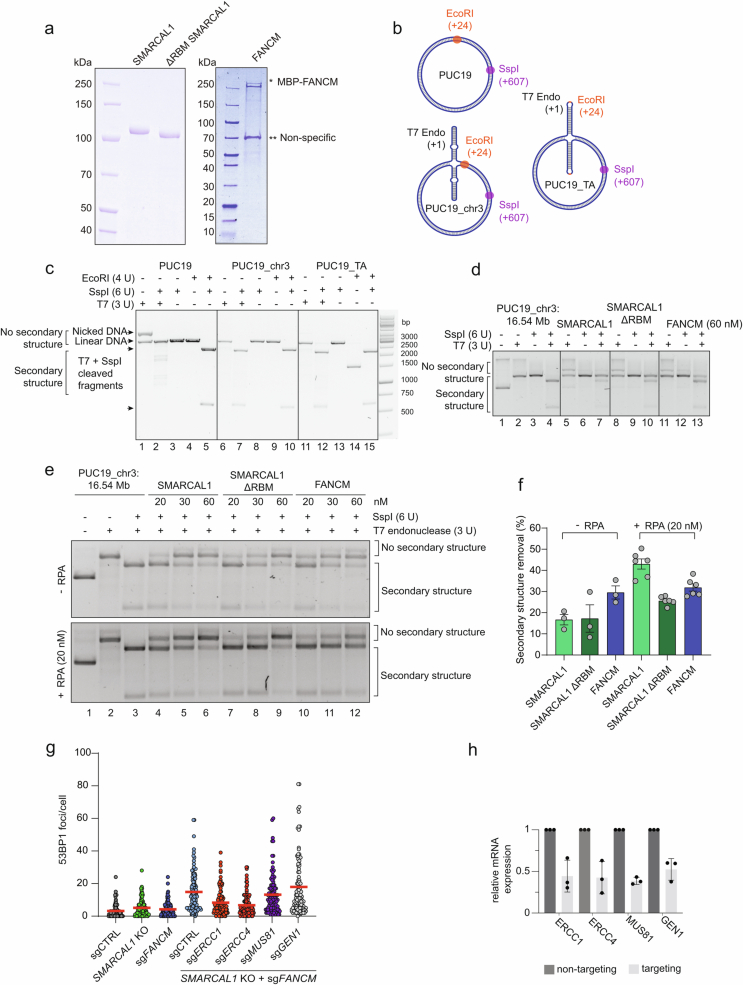
Related to Fig. 4. In vitro cruciform unwinding and rescue of 53BP1 foci. **a**, Polyacrylamide gels showing recombinant SMARCAL1, SMARCAL1 ΔRBM and FANCM (*n* = 1). **b**, Schematic of the different substrates used in the unwinding assays. **c**, Gels validating secondary structure formation. Incubation with the structure-specific T7 Endonuclease I and SspI results in the production of bands at the expected positions of 606 and 2,120 bp (*n* = 1). The SspI restriction site is 606 bp away from the cruciform site. Incubation with EcoRI produces a linear DNA fragment with PUC19 and PUC19_chr3. On PUC19_TA, the EcoRI sites are at the apex of the cruciform, and hence refractory to cleavage when a cruciform is present. **d**, Representative cruciform unfolding assay on the PUC19_chr3 substrate showing SMARCAL1, SMARCAL1 ΔRBM and FANCM prevent DNA cutting by T7 endonuclease I (*n* = 2). **e**, Representative unwinding assay of PUC19_chr3 in the presence and absence of RPA (20 nM). SMARCAL1 ΔRBM shows a reduced activity in the presence of RPA at lower concentrations. Quantified for 30 nM in **f. g**, Quantification of 53BP1 foci in the indicated cells. At least 100 cells were quantified per condition. Red line denotes the mean. **h**, RT-qPCR data of cells transduced with sg*CTRL*, sg*ERCC1*, sg*ERCC4*, sg*MUS81*, sg*GEN1* show knockdown of mRNA transcripts for each sgRNA. Error bars represent mean ± s.d. of three experimental replicates. Source data

To address whether FANCM and SMARCAL1 can directly unwind the TA cruciforms observed in cells, we turned to biochemical experiments using recombinant proteins and purified DNA substrates (Extended Data Fig. 12a–c). SMARCAL1 and FANCM were individually capable of remodelling both a pure-TA cruciform and an interrupted TA repeat that was bound by FANCM and SMARCAL1 in double-perturbed cells (Fig. 4c and Extended Data Fig. 12c–f). In the presence of RPA, wild-type SMARCAL1 was much more efficient than SMARCAL1 ΔRBM at suppressing cruciforms (Extended Data Fig. 12e,f). These data directly demonstrate the activity of FANCM and SMARCAL1 on TA-rich cruciforms.

Cruciforms can be inappropriately cleaved to DSBs by endonucleases, as is the case for the MUS81 nuclease at expanded TA repeats in WRN-depleted microsatellite-unstable cancers^7^. Notably, WRN depletion is not synthetic lethal with either FANCM or SMARCAL1 depletion in RPE-1 cells, and our data suggest that WRN does not readily compensate for the combined loss of FANCM and SMARCAL1. Testing a panel of structure-specific nucleases, we found that depletion of MUS81 impacted MRE11 enrichment at a subset of sites but had little effect on 53BP1 foci formation (Fig. 4d,e and Extended Data Fig. 12g,h). However, depletion of the ERCC1–ERCC4 nuclease complex significantly reduced the number of 53BP1 foci and MRE11 enrichment at TA repeats in SMARCAL1:FANCM-deficient cells (Fig. 4d,e and Extended Data Fig. 12g,h).

Our data support a model in which the complementary activities of FANCM and SMARCAL1 remodel cruciforms that form at TA-rich repeats (Fig. 4f). The absence of both translocases leads to cruciform persistence and their inappropriate cleavage, predominantly by the ERCC1–ERCC4 complex. Such cleavage events evade repair in late-replicating regions, or the cruciforms themselves may be structural impediments to replication, ultimately causing cell death (Fig. 4f).

## Discussion

Using a dual-guide CRISPRi screen, we queried approximately 150,000 genetic interactions between 548 core DDR genes, encompassing all major DNA repair pathways and including essential genes. These experiments were performed in unperturbed cells with an intact DDR, yielding new insights into the repair processes required for cells to survive everyday DNA transactions. We identified over 5,000 high-confidence interactions, recapitulating known interactions and implicating new factors in diverse processes. Mechanistically, we focused on two of the strongest synthetic lethal gene pairs identified in our screen: *FEN1*/*LIG1*:*WDR48–USP1* and *FANCM*:*SMARCAL1*.

We found that the USP1-activating function of WDR48 counteracts RAD18 ubiquitin ligase activity to prevent PCNA degradation when ssDNA gaps accumulate. We propose that PCNA degradation drives cell death by causing DNA under-replication, leading to genome instability and chromosome breakage. Approximately 30–50 million Okazaki fragments are formed during each S phase, and single-strand break repair proteins, such as PARP1, LIG3 and XRCC1, are required to deal with fragments that are inevitably missed by FEN1 and LIG1 (refs. ^21,54^). Our study implies a crucial role for WDR48–USP1 in preventing DNA gaps from inducing PCNA hyper-ubiquitylation and subsequent cell death during unperturbed DNA replication.

Our complementation data suggest that the paucity of PCNA, and not TLS activation, drives cell death. However, PCNA ubiquitylation could affect activities, such as PCNA unloading or template switching^55^. Toxic hyper-ubiquitylation of PCNA was previously reported upon USP1 inhibition in BRCA1-deficient cells^56^. The profound sensitivity of FEN1-deficient cells to the USP1 inhibitor KSQ-4279 also suggests it might be effective in treating *FEN1*-mutant cancers or augmenting chemotherapies that induce DNA gap formation, such as ATR and WEE1 inhibitors^41^. This is further supported by the synergy of USP1 and PARP or POLQ inhibitors in BRCA1-deficient backgrounds^57^.

The identification of a genetic interaction between *FANCM* and *SMARCAL1* revealed complementary roles for these factors in disrupting DNA cruciforms at long, late-replicating TA-rich repeats. While this study was under revision, the Durocher lab also reported a profound synthetic lethality between FANCM and SMARCAL1 (ref. ^58^). Whether TA-rich cruciforms contribute to cell death by acting as structural barriers to DNA replication is unclear. However, our data strongly suggest that when both SMARCAL1 and FANCM are absent, cruciforms render the genome vulnerable to cleavage predominantly by the ERCC1–ERCC4 complex.

These data correspond with the preference of ERCC1–ERCC4 for cleaving cruciform structures^59–61^. Cells then enter mitosis with broken chromosomes, and mitotic catastrophe ensues. Our findings have parallels to the essential role of the WRN helicase at expanded TA repeats in microsatellite-unstable cancers and suggest several layers of surveillance against dangerous DNA structures during every cell division. *FANCM* is frequently mutated in several cancers, including triple-negative breast cancers, highlighting SMARCAL1 as a potential drug target^62^. FANCM may also represent a target for therapeutic intervention in cancers with loss-of-function *SMARCAL1* mutations, which are prevalent in glioblastomas^63^.

Our DDR interaction map identifies numerous synthetic lethal relationships that could be exploited in cancer therapy. Many already link established cancer genes and existing small-molecule targets and thus are readily actionable. We anticipate that exploration of our dataset will define more cancer-specific vulnerabilities that could translate into clinical benefit in patients.

## Methods

### Cell culture

The hTERT RPE-1, HEK293(T) and HeLa S3 cell lines were cultured in Dulbecco’s modified Eagle’s medium/F12 (Merck) supplemented with 10% fetal bovine serum (FBS; Thermo Fisher Scientific) and 100 U ml^−1^ of streptomycin and 100 mg ml^−1^ of penicillin (Gibco). HeLa S3 dCas9–ZIM3 cells were cultured in the presence of 200 µg ml^−1^ of hygromycin to maintain selection. K562 dCas9–KRAB cells were cultured in RPMI-1640 with 25 mM HEPES and 2.0 g l^−1^ of NaHCO_3_ in 10% FBS, 2 mM glutamine, 100 U ml^−1^ of streptomycin and 100 mg ml^−1^ of penicillin. All cell lines were grown under 3% oxygen. Cells were routinely tested for mycoplasma.

### Cell line generation

The hTERT RPE-1 *TP53* KO cells were obtained from the Stephen Jackson lab (Cambridge). RPE-1 *TP53* KO dCas9–KRAB cells were generated by transduction with a lentiviral vector encoding the dCas9–KRAB and a blasticidin resistance cassette with a low multiplicity of infection (MOI). Cells were selected using blasticidin, and single-cell clones were seeded by cell sorting. The resulting clones were validated for CRISPRi activity by CD55 knockdown efficiency. RPE-1 *TP53* KO, *FANCM* KO and *SMARCAL1* KO cell lines were generated using CRISPR–Cas9. SgRNAs were selected from the Vienna BioCenter (VBC) website and were in vitro transcribed with T7 RNA polymerase^64^. Editing of cells was done, as described previously^65^. A Lonza Amaxa nucleofector was used with P3 solution and program EA104. Clones were single-cell seeded by serial dilution, and clones were selected using Sanger Sequencing and ICE analysis (Synthego). KO of *FANCM* and *SMARCAL1* was validated by next-generation sequencing and western blotting. A pool of HeLa S3 dCas9–ZIM3-expressing cells was generated by transduction with a lentiviral vector encoding dCas9–ZIM3 and a hygromycin resistance cassette, followed by selection with 200 µg ml^−1^ of hygromycin.

### Cloning and mutagenesis

For RNase H1 wild-type or WKKD mutant (W43A, K59A and K60A), cDNAs from Addgene plasmid nos. 111906 and 111905 were polymerase chain reaction (PCR) amplified. The resulting fragments and mCherry cDNA amplified from Addgene plasmid no. 102245 were assembled using Gibson assembly. Empty and *FANCM* (wild type and K117R) pLVX-TetOne-Puro plasmids were a generous gift from the Claus Maria Azzalin Lab^66^. pAcGFP-C1–RPA123–P2A was a generous gift from the Jiri Lukas laboratory. Point mutants were created by site-directed mutagenesis. Internal deletion mutants were generated by inverse PCR, followed by T4 polynucleotide kinase phosphorylation and blunt-end ligation. 3×Flag–*SMARCAL1*, *PCNA*, *FEN1* and *LIG1* cDNAs were cloned into the lentiviral vector pLVX-TetOne-Puro (Clontech) using Gibson assembly. For all Gibson assemblies, the New England Biolabs (NEB) HiFi Assembly Master Mix was used. All PCR reactions were performed using Q5 DNA Polymerase (NEB).

### Guide RNA cloning

CRISPRi sgRNAs were cloned into either BFP-, GFP- (Addgene plasmid nos. 60955 and 111596) or mCherry-containing lentiviral vectors or, for dual-guide expression, into pJR103 (Addgene plasmid no. 187242). A neomycin resistance cassette-containing lentiviral vector was used for sgRNAs targeting *RAD18* (Fig. 2c,d,f,g and Extended Data Figs. 5h,i, 6e–f and 7a,b,e–g), *LIG1* (Fig. 2e and Extended Data Fig. 7d,h), and *FANCM* (Figs. 3a and 4d,e and Extended Data Fig. 12g). For dual guide expression vectors, homologies with the backbone and protospacers were added to the insert from pJR98 (Addgene plasmid no. 187239; containing the constant region CR3 for the first sgRNA and hU6 promoter for the second sgRNA) using PCR. PCR products were assembled with JR103 using Gibson assembly. Forward and reverse oligos for each sgRNA were annealed by preincubation at 37 °C for 30 min with T4 polynucleotide kinase (NEB), followed by incubation at 95 °C for 5 min and then ramp down to 25 °C at 5 °C min^−1^. Annealed sgRNAs were ligated into the corresponding vector that had been digested with BstXI and BlpI restriction enzymes using T4 ligase (NEB). All sgRNA protospacers are listed in Supplementary Table 6.

### Design of double-sgRNA CRISPRi SPIDR library

The genes to target in the SPIDR sgRNA library (*n* = 548) were selected by all genes with the gene ontology term ‘DNA repair’ (GO:0006281). For each gene, sgRNAs from the human CRISPRi-v2 library were ranked using a strategy described previously and a preliminary dataset of more than 50 CRISPRi screens^10,12^. Briefly, the genes were divided into three tiers. For genes essential for growth in K562 cells (tier 1), sgRNAs were ranked according to their growth phenotypes. For genes that had scored as significant hits in at least four CRISPRi screens (tier 2), sgRNAs were ranked according to their average phenotype across all screens in which the genes had scored as hits. For all other genes (tier 3), sgRNAs were ranked according to the regression scores from the human CRISPRi v.2.1 algorithm. For each gene, two sgRNAs were then selected for each transcription start site targeted in the human CRISPRi-v2 library, as follows.

For genes (*n* = 249) for which KO caused strong growth phenotypes in either RPE-1 cells or in at least five neuroblastoma cell lines, as assessed by querying data from 17 screens in neuroblastoma cell lines (identifiers NB5, NB6, NB7, NB10, NB13, NB17, NB69, CHP-212, SK-N-FI, SK-N-AS, SK-N-DZ, SK-N-SH, KP-N-YN, KP-N-YS, TGW, BE2-M17 and NH-12) in the Project Score database from the Cancer Dependency Map (https://score.depmap.sanger.ac.uk/, accessed August 2020), these two sgRNAs were (1) the top sgRNA identified using the algorithm above and (2) a mismatched variant of the top sgRNA, chosen to have an empirical relative activity of 0.47 ± 0.15 if an sgRNA existed for which the relative activity in this window had been measured or otherwise chosen as the mismatched variant with predicted activity closest to 0.5 out of all possible singly mismatched variants, with activity predictions derived from a convolutional neural network, as previously described^11,67,68^. Mismatched sgRNAs were required to have an on-target specificity score of at least 0.15, calculated as described previously^11^.

For genes (*n* = 299) for which KO did not cause strong growth phenotypes in both RPE-1 and more than 12 neuroblastoma cell lines, the top 2 sgRNAs from the algorithm above were selected.

From this set of sgRNAs, the double-sgRNA libraries were assembled in a programmed manner. Each unique combination of two genes was randomly assigned an orientation of ‘ab’ or ‘ba’. For each such a combination, all sgRNAs targeting the gene in position a were paired with all sgRNAs targeting the gene in position b. Each sgRNA targeting a given gene was also paired with all other sgRNAs targeting that gene. Each unique sgRNA in the library was furthermore paired with 15 different non-targeting negative control sgRNAs in both the ab and ba orientations. Finally, 225 pairs consisting of all possible combinations of 15 different non-targeting negative control sgRNAs were included as negative controls.

All sgRNA pairs were then ordered as an oligo pool from Agilent Technologies, containing the following constant sequences: AACTGCGATCGCTAATGTCCACCTTGTTG (upstream of sgRNA a), gtttcagagcgagacgtgcctgcaggatacgtctcagaaacatg (between sgRNA a and sgRNA b) and GTTTAAGAGCTAAGCTGGTTCTCCAGTGCCTTATT (downstream of sgRNA b).

### Dual-guide library cloning

Dual-guide library cloning was performed, as described in a previous study^12^ with small modifications. After PCR amplification, oligos were BstXI/BlpI-digested and run on a polyacrylamide gel. The corresponding band was extracted, and DNA was purified before ligation into pJR103. The ligated DNA was precipitated using isopropanol (catalogue no. 17170576; Thermo Fisher Scientific) and transformed into MegaX Electrocomp Cells (catalogue no. C640003; Thermo Fisher Scientific). Bacteria were cultured in lysogeny broth overnight, and plasmid was collected with Plasmid Plus Midi Kit (QIAGEN). This intermediate library and pJR98 were digested using BsmBI, and the insert from pJR98 was ligated into the intermediate library to insert constant region CR3 for the first sgRNA and hU6 promoter for the second sgRNA. A library coverage of at least 30 times was continually maintained during the cloning process. The final library expresses the first sgRNA under the mU6 promoter with CR3, whereas the second sgRNA is expressed under a hU6 promoter with CR1.

### Lentivirus packaging

Lentiviruses were produced in HEK293T cells. Briefly, HEK293T cells were transfected using polyethylenimine with the VSV-G envelope expressing plasmid (pMD2.G), packaging plasmid (psPAX2) and our transfer vector. Lentiviral supernatant was collected 48 and 72 h after transfection.

### CRISPRi screens

The hTERT RPE-1 *TP53* KO cells stably expressing dCas9–KRAB were transduced with the lentiviral library at an MOI of approximately 0.25 with a coverage of at least 350 cells per sgRNA in a medium supplemented with polybrene (10 μg ml^−1^). The next day, the culture medium was replaced with a puromycin-containing medium (15 μg ml^−1^) to select for transductants. Selection was carried out for 96 h, during which time cells were expanded and divided into two biological replicates. Hereafter, library coverage was maintained at approximately 500 cells per sgRNA combination. Four days after transduction, the background/time point 0 samples were collected and cell pellets were frozen (−80 °C). The remaining cells were seeded and continually subcultured when near 100% confluency for 14 days (approximately ten population doublings), at which time cell pellets were collected and frozen (−80 °C). Genomic DNA was isolated using the Gentra Puregene Cell Kit (QIAGEN). Integrated sgRNA regions were amplified by PCR using custom primers (Supplementary Table 7), and paired-end sequencing was performed on a NovaSeq 6000. Screens in HeLa S3 and K562 cells were performed, as described above, with the following modifications. Transduced HeLa S3 dCas–ZIM3 and K562 dCas9–KRAB cells were selected in a medium containing 1 or 5 μg ml^−1^ of puromycin, respectively, and selection was initiated 48 h after lentiviral transduction. HeLa S3 dCas9–ZIM3 cells were cultured in the presence of 200 µg ml^−1^ of hygromycin for the entirety of the screen.

### Screen analysis

Raw FASTQ files were processed using seal (BBMap; B. Bushnell; sourceforge.net/projects/bbmap/) to search for sgRNA matches in the first 22 bp of each read with a hamming distance of 0 and a *k*-mer length of 20 bp. A custom Python script was used to parse the annotated FASTQ files and generate a count matrix containing all possible sgRNA pairs. The coverage per sgRNA pair was calculated to assess the quality of each sample (Extended Data Fig. 1a,b). Total counts were normalized using the median ratio method to estimate size factors using the non-targeting sgRNAs^69^. R v.4.1.2 was used to generate custom plots. Normalized sgRNA counts are provided in Supplementary Table 8.

Normalized counts were used to obtain log-fold changes (LFCs) from the GEMINI analysis. Count data were filtered with having a representation of 50 or more reads in the T0 sample of each replicate. LFCs were calculated using the GEMINI package with a pseudo-count of 10 and a modified gemini_calculate_lfc_mod function to prevent double normalization (see Supplementary Information). Essential sgRNAs were determined with having an average LFC of less than −3 at T14 when combined with non-targeting sgRNAs. Perfect and mismatched variants of these sgRNAs were included as separate genes in the analysis for Extended Data Fig. 2c to determine the recovery of genetic interactions for strong essential genes. Mismatched variants of strong essential sgRNAs showed a reduced signal at T14 and were able to recover more genetic interactions (Extended Data Fig. 2a–c). Therefore, count data were filtered for these candidates, and only the mismatched sgRNA variant was kept for all analysis. For HeLa S3 and K562, essential genes were defined with the same *z*-score cut-off as for RPE-1 LFC less than −3. Non-targeting sgRNAs were used as the negative control gene to build the GEMINI model. Sensitive and strong GEMINI scores were calculated with a scaling factor *λ* of 1 to identify synthetic lethal interactions of a broad range of genes. GEMINI scores greater than 0 were considered potential genetic interactions, and all combinations with a score of less than 0 were considered non-interacting and assigned a score of 0. The resulting GEMINI scores were given negative signs. For the genetic interaction heat map, the full SPIDR T14 dataset was filtered for genes having interactions with at least one other gene with a GEMINI sensitive score of 2 s.d. or more away from the mean non-zero score. Clusters were generated using agglomerative clustering with Ward variance minimization. Pairwise and higher physical interactions within each cluster were retrieved from STRING v.12 with a confidence cut-off of 0.4. UMAP embedding was calculated in R using the strong score across a symmetric version of the entire dataset, without prior dimension or variance reduction. A Venn diagram for the focused SPIDR screens was created using R with a GEMINI score cut-off of −1 or less for K562 cells and −0.5 or less for HeLa S3 cells.

### Dual-colour flow cytometry assay

The indicated cells were transduced with sgRNA-containing lentiviruses at the same MOI. After 72 h, the cells were seeded in triplicate in six-well plates, and after an extra 24 h, the cell populations were analysed by flow cytometry (day 0) using an Attune NxT Flow Cytometer (Invitrogen). The cell populations were analysed at regular time intervals (as indicated in the figures) thereafter. For triple knockdown and cDNA experiments, cells were transduced with the relevant sgRNA-containing lentiviruses and selected with puromycin for 96 h before transduction with lentiviruses containing sgRNAs targeting synthetic lethal gene pairs. For cDNA experiments, expression was induced with 0.25–1 μg ml^−1^ of DOX 24 h before transduction with lentiviruses containing sgRNAs. The DOX-containing medium was refreshed every 48 h. Where drug treatments were performed, cells were treated 24 h after seeding on six-well plates (on day 0). All values were normalized to the corresponding untransduced control cells.

### Clonogenic survival assays

The hTERT RPE-1 *TP53* KO cells were co-transduced with lentiviruses containing sgRNAs that were co-expressed with either a puromycin or neomycin resistance cassette. After selection with puromycin (10 μg ml^−1^) and G418 (1.25 mg ml^−1^), 1,000 cells of each sample were seeded in triplicate on six-well plates. Where indicated, drug treatments were performed 24 h after seeding. Ten days later, cells were rinsed in PBS and stained with 0.5% (w/v) crystal violet in 20% (v/v) methanol for 15 min at room temperature. After staining, the plates were rinsed with H_2_O and air-dried.

### Competitive growth assays

Cells were independently transduced with either a non-targeting sgRNA (co-expressed with either mCherry, GFP or BFP) or a targeting sgRNA. After 24 h, the cells were treated with the appropriate antibiotic, either puromycin (10 μg ml^−1^) or G418 (1.25 mg ml^−1^), until untransduced cells were selected from the population. The cells were then mixed at a 1:1 ratio by number and seeded in triplicate on six-well plates. After 24 h, cell populations were analysed using flow cytometry. The cells were either treated or not with exogenous agents, as indicated in the figures, and monitored using flow cytometry at the indicated time points thereafter.

### Drug treatments

The chemicals used in this study can be found in Supplementary Table 7. Treatment durations and doses are indicated in the figures/legends.

### Reverse transcription–quantitative PCR

One million RPE-1 cells were collected, and total RNA was isolated using the RNeasy Kits (QIAGEN), according to the manufacturer’s instructions. RNA was reverse transcribed into cDNA using iScript Reverse Transcription Supermix (Bio-Rad) using oligo(dT) primers. Quantitative PCR (qPCR) reactions were prepared with the SsoAdvanced Universal SYBR Green Supermix (Bio-Rad) and ran on a QuantStudio 6 Flex Real-Time PCR System (Applied Biosystems). Primers used for qPCR can be found in Supplementary Table 7. Data were analysed using the Delta Delta Ct (ΔΔCt) method.

### Nucleofection of RNase H1 constructs

For transient expression of RNase H1 constructs, 100,000 RPE-1 cells were transfected with 1 μg of RNase H1 plasmid using the Lonza Amaxa nucleofector with P3 solution and program EA104. Nucleofected cells were recovered in a warm medium and identified by mCherry expression using flow cytometry. All experiments with RNase H1 variants were carried out by gating only for the mCherry-positive cells.

### Cell cycle analysis

The hTERT RPE-1 *TP53* KO cells were arrested in G0/G1 by contact inhibition for 48 h and then re-plated at low density (100,000 cells per well on a six-well plate) to release them into S phase in the presence or absence of ML323 (30 μM). The cells were collected at the indicated time points, fixed in 70% ethanol for 30 min on ice, washed once with staining buffer (1× PBS + 5% FBS) and stained at room temperature using 1 µg ml^−1^ of 4′,6-diamidino-2-phenylindole (DAPI) solution (BD Biosciences) diluted in staining buffer for 5 min. For EdU–DAPI assay, 100,000 cells per well were seeded in a medium containing 1 µg ml^−1^ DOX. On the following day, the cells were treated with KSQ-4279 (25 μM) for 48 h and incubated with 10 μM EdU for the last 1 h before collecting. Click-iT Plus EdU Alexa Fluor 488 Flow Cytometry Assay Kit (Thermo Fisher Scientific) was used according to the manufacturer's instructions, and the cells were resuspended in 1 µg ml^−1^ DAPI solution (BD Biosciences) diluted in 1× Click-iT permeabilization and wash reagent for 5 min. The cells were analysed using an Attune NxT Flow Cytometer. Subsequent analysis was performed using FlowJo v.10.8.1.

### Western blotting

Cells were lysed in radioimmunoprecipitation assay buffer (0.5 M Tris-HCl (pH 7.4), 1.5 M NaCl, 2.5% deoxycholic acid, 10% NP-40 and 10 mM EDTA), supplemented with Halt Protease Inhibitor Cocktail and Phosphatase Inhibitor Cocktail (Thermo Fisher Scientific). Samples were sonicated using a Bioruptor Plus sonicator (30 s ON and 30 s OFF for five cycles) and centrifuged for 5 min at 21,000*g*. Protein concentrations were measured using Bradford assay. The samples were normalized by protein concentration, mixed with 4× NuPAGE LDS Sample Buffer supplemented with 5% β-mercaptoethanol and boiled for 5 min at 98 °C. The samples were loaded into Bolt 4–12% Bis-Tris Protein Gels (Thermo Fisher Scientific) and transferred to 0.2-μm nitrocellulose membranes. Membrane blocking was performed with 5% milk diluted in Tris-buffered saline containing 0.1% Tween 20. Primary antibodies were diluted in 5% bovine serum albumin (BSA) diluted in Tris-buffered saline containing 0.1% Tween 20. After incubation with primary antibodies, membranes were incubated with Li-Cor near-infrared fluorescence or HRP secondary antibodies, after which they were scanned using a Li-Cor Near-InfraRed fluorescence Odyssey CLx Imaging System or FujiFilm LAS 4000 Gel Imager. All antibodies used are listed in Supplementary Table 7. All primary antibodies were used at a dilution of 1/1,000. All secondary antibodies were used at a dilution of 1/10,000.

### Immunohistochemistry

Cells were seeded 48 h before on sterile 15-mm glass coverslips. The coverslips were washed in ice-cold PBS, and nuclei were 5 min pre-extracted by incubating cells in a pre-extraction buffer (HEPES (pH 7.5; 25 mM), NaCl (50 mM), EDTA (1 mM), MgCl_2_ (3 mM), sucrose (300 mM) and Triton X-100 (0.5%)) on ice. The cells were washed once in PBS and then fixed in 4% formaldehyde for 15 min at room temperature. The coverslips were again washed two times in PBS before blocking in 5% BSA in PBS ON at 4 °C. Primary antibody incubation with 53BP1 antibody (NB100-304) was carried out ON at 4 °C. After three washes in PBS, the coverslips were incubated with the secondary Alexa Fluor 488 antibody for 1 h at room temperature. The coverslips were washed three times in PBS and then incubated with Hoechst 33342 Ready Flow Reagent for 5 min before two more wash steps in PBS and a Milli-Q water wash. Cells were mounted onto glass slides using ProLong Diamond Antifade Mountant. All antibodies were diluted in 2.5% BSA in PBS. All incubation steps were carried out in a wet chamber. Images were taken using a Leica SP8 confocal microscope or ZEISS Apotome 3. For foci analysis, the layer with the highest intensity was selected. If there were foci in several planes, *z*-stack and maximum intensity projection were performed. For quantification of mitotic catastrophes, Hoechst staining was used to detect nuclear morphology, and fragmented nuclei were scored, as shown in Fig. 3e. The data were quantified using Fiji ImageJ v.2.9.0.

To detect 53BP1 nuclear bodies, cells were grown on a chambered coverslip for 24 h before being washed in PBS and fixed in 4% formaldehyde for 10 min at room temperature. The cells were washed three times in PBS and permeabilized with 0.5% Triton X-100 in cytoskeleton buffer (100 mM NaCl, 20 mM HEPES (pH 7.0), 3 mM MgCl_2_ and 300 mM sucrose) for 10 min at 4 °C. The cells were washed three times with PBS and blocked with 5% BSA (diluted in PBS) for 30 min. The cells were then incubated with primary antibodies (cyclin A, 1:200; 53BP1, 1:2,000) for 90 min at room temperature, washed three times in PBS and then stained with secondary antibodies (both 1:1,000) for an extra 45 min. ProLong Gold mounting reagent with DAPI was added to each coverslip. The cells were imaged using a Leica SP8 confocal microscope or ZEISS Apotome 3. Nuclear bodies in cyclin A-negative cells were manually counted. Representative images were prepared using QuickFigures ImageJ plugin^70^.

To detect ssDNA using BrdU labelling under native conditions, cells were grown on a chambered coverslip, incubated with 10 μM BrdU for 72 h and then pre-extracted, as described above. The cells were incubated with primary antibodies (BrdU, 1:250; RPA32, 1:500) ON and then stained with secondary antibodies (both 1:1,000) for 45 min. An ibidi mounting medium containing DAPI was used, and cells were imaged using a ZEISS Apotome 3. BrdU- and RPA32-positive foci were considered ssDNA stretches and were manually counted.

### Metaphase spreads

Cells were treated with 0.04 μg ml^−1^ of colcemid for 16 h to arrest them in mitosis and then collected, washed with PBS and incubated in 0.075 M KCl at 37 °C for 10 min. Cell pellets were fixed in methanol:acetic acid (3:1 ratio), spread on glass slides and then coated with ProLong Diamond Antifade Mountant. Spreads were imaged using a ZEISS Apotome 3 microscope.

### S1 nuclease DNA fibre assays

The S1 nuclease DNA fibre assay was conducted, as described in a previous study^71^, with the following modifications. Cells were incubated with 19 mM CldU for 20 min, washed three times with warm PBS and then incubated with 28 mM IdU for 60 min. During the IdU incubation, the cells were either treated or not with KSQ-4279 (25 μM). The cells were then collected and incubated with cytoskeleton-100 buffer (100 mM NaCl, 20 mM HEPES, 3 mM MgCl_2_, 300 mM sucrose and 0.5% Triton X-100) for 5 min and then washed with PBS. The cells were then incubated in 200-μl S1 nuclease reaction buffer with or without S1 nuclease (20 U ml^−1^; Thermo Fisher Scientific) for 30 min at 37 °C. The cells were then washed with PBS, lysed on glass slides in a buffer composed of 200 mM Tris-HCl (pH 7.5), 50 mM EDTA and 0.5% sodium dodecyl sulfate (SDS) for 5 min, and spread by tilting the slides at a 45° angle. The slides were then air dried and fixed in methanol:acetic acid (3:1 ratio) overnight at 4 °C. The slides were then incubated in 2.5 M HCl for 1 h to denature the DNA fibres, washed with PBS and incubated in 1% BSA/PBS (0.2% Tween 20) for 40 min. Staining was performed by incubating the slides for 2.5 h at room temperature with anti-CldU (1:500; ab6326; Abcam) and IdU (1:100; B44; 347,580; BD Biosciences) antibodies, followed by 1-h room temperature incubation with anti-mouse Alexa Fluor 488 (1:300) and anti-rat Alexa Fluor 568 (1:150) secondary antibodies. All antibodies were diluted in 2.5% BSA/PBS. Fibres were visualized using a Leica SP8 confocal microscope or ZEISS Apotome 3 microscope (×64; oil) and analysed using Fiji ImageJ v.2.9.0.

### Live imaging

Cells were grown on chambered coverslips. PIP–FUCCI reporter-expressing cells were seeded into a medium containing 50–100 nM palbociclib. Twenty-four hours later, the medium was replaced with Dulbecco’s modified Eagle’s medium (no phenol red) supplemented with either dimethyl sulfoxide (DMSO) or KSQ-4279 (20 μM). For H2B–GFP experiments, cells were treated with DMSO or KSQ-4279 (25 μM) for 24 h before being imaged for an extra 24 h. All live cell imaging experiments were performed using a Nikon spinning disk equipped with a Yokogawa Confocal Scanner Unit CSU-W1-T2 SoRa at ×20 magnification.

### ChIP–seq

For each condition, 10–20 million cells were fixed in 1% formaldehyde at room temperature for 15 min. The fixation reaction was quenched with glycine to a final concentration of 125 mM. Cells were collected and washed twice with chilled PBS, and pellets were snap frozen in a dry ice–ethanol bath before storing at −80 °C. When ready to process, cell pellets were thawed on ice and incubated with lysis buffer (LB)1 (50 mM HEPES–KOH (pH 7.5), 140 mM NaCl, 1 mM EDTA, 10% glycerol, 0.5% NP-40 or Igepal CA-630, 0.25% Triton X-100 and 1× protease inhibitors) on ice for 10 min. Cells were pelleted by centrifugation and incubated with LB2 (10 mM Tris-HCl (pH 8.0), 200 mM NaCl, 1 and 0.5 mM EDTA and 1× protease inhibitors) for 5 min on ice. The extracted nuclei were pelleted by centrifugation and resuspended in LB3 (10 mM Tris-HCl (pH 8.0), 100 mM NaCl, 1 mM EDTA, 0.1% Na deoxycholate, 0.5% N-lauroylsarcosine and 1× protease inhibitors). Nuclei were sonicated using a Covaris S2 sonicator with the following settings: duty cycle 5%, intensity 5, 200 cycles per burst and 12 min. Debris was pelleted by centrifugation at 4 °C, and the supernatant was transferred to a 5-ml tube. Then, 100 µl of Dynabeads Protein A that had been prebound with MRE11 (catalogue no. NB100-142; Novus), FANCM, SMARCAL1 or DNA cruciform antibody was added to the cell lysate, and samples were incubated at 4 °C overnight with rotation. Beads were collected on a magnetic stand and washed with ice-cold radioimmunoprecipitation assay buffer six times, followed by a final wash with Tris-buffered saline before resuspending beads in 200 µl of elution buffer (50 mM Tris-HCl (pH 8.0), 10 mM EDTA and 1% SDS). The bead slurries were incubated overnight at 65 °C to reverse crosslinks. Samples were treated with 1 mg ml^−1^ RNaseA (catalogue no. 2271; Ambion) for 30 min at 37 °C, followed by proteinase K treatment (20 mg ml^−1^; catalogue no. 25530-049; Invitrogen) for 1 h at 55 °C. DNA was then purified using a MinElute PCR Purification Kit (catalogue no. 28004; QIAGEN), and sequencing libraries were prepared using a NEBNext Ultra II kit.

### ChIP–seq analysis

Raw paired-end FASTQ files were aligned to the GRCh38 reference genome using Bowtie 2 v.2.4.4. The resulting BAM files were sorted and indexed with Samtools v.1.6. Peaks were called using MACS3 v.3.0.0b1 with RPE-1 wild type set as the control and default settings. Peaks were filtered using the ENCODE blacklist v2 and manually inspected^72^. Total read counts were calculated for each sample and used to generate comparable bigWig files for visualization using bamCoverage v.3.3.0 and ScaleFactor option. CrossMap v.0.6.0 was used to lift over GRCh38 to GRCh19 coordinates. Locally hosted bigWig files were added as custom tracks to the UCSC Genome Browser for visual inspection. MEME-ChIP v.5.5.1 was used to identify motifs around the peaks, and ChIPseeker was used to visualize peak locations over chromosomes. The AT fraction was calculated per base across all peaks in a 1,000-bp window around the centre of each peak. In Fig. 4e, MRE11 enrichment was determined at a union set of TA repeat sites (more than 70% TA content) consisting of FANCM peaks in *SMARCAL1* KO cells, SMARCAL1 peaks in *FANCM* KO cells and unique cruciform and MRE11 peaks in double-perturbed cells.

### Repli-seq analysis

Repli-seq data for GM06990 cells from the University of Washington Encyclopedia of DNA Elements group were downloaded from the UCSC Genome Browser as bigWig files in wavelet-smoothed and percentage-normalized signal format (GEO accession: GSM923443). DeepTools v.3.5.1 was used to visualize the wavelet-smoothed signal data for all peaks in a 2-Mb region around each peak centre with the tools computeMatrix reference-point, the bin-size option set to 1 and plotHeatmap to create the heat map. R was used to process and plot the percentage-normalized signal data.

### Protein purification

#### Human SMARCAL1

The hSMARCAL1 wild-type and ΔRBM variants were expressed in *Spodoptera frugiperda* 9 (*Sf*9) cells using the Bac-to-Bac expression system (Thermo Fisher Scientific), according to the manufacturer’s recommendations. The protein sequences were codon optimized for the expression in *Sf*9 insect cells and cloned NheI and XhoI restriction sites of pFastBac1, generating pFB-2xMBP-hSMARCAL1-10xHis. Frozen *Sf*9 pellets from 200-ml cultures for each variant were resuspended in LB (50 mM Tris-HCl (pH 7.5), 5 mM β-mercaptoethanol, 1 mM phenylmethanesulfonylfluoride (PMSF), 1 mM EDTA, protease inhibitor cocktail (P8340; Sigma-Aldrich) diluted 1:300 and 30 µg ml^−1^ of leupeptin (Merck Millipore)) and incubated at 4 °C for 20 min. Glycerol was added to a final concentration of 16.7%. NaCl was added to a final concentration of 305 mM. The solution was incubated at 4 °C for 30 min. The mixture was centrifuged at 20,000*g* at 4 °C for 30 min. The resulting soluble extract was incubated with 4-ml amylose resin (NEB) at 4 °C for 1 h. The resin was washed with amylose wash buffer 1 M (50 mM Tris-HCl (pH 7.5), 5 mM β-mercaptoethanol, 1 mM PMSF, 10% glycerol and 1 M NaCl) followed by 300 mM amylose wash buffer (50 mM Tris-HCl (pH 7.5), 5 mM β-mercaptoethanol, 1 mM PMSF, 10% glycerol and 300 mM NaCl). Proteins were eluted using amylose elution buffer (50 mM Tris-HCl (pH 7.5), 5 mM β-mercaptoethanol, 1 mM PMSF, 10% glycerol, 300 mM NaCl and 10 mM maltose). The MBP-tagged variants were incubated with PreScission Protease (approximately 10 μg of PreScission protease per 100 μg of tagged protein) at 4 °C for 1 h to cleave the MBP tag. Subsequently, the cleaved protein was diluted 1:3 to reduce the concentration of NaCl to approximately 100 mM with a buffer containing no NaCl (50 mM Tris-HCl (pH 7.5), 5 mM β-mercaptoethanol, 1 mM PMSF and 10% glycerol) and applied in flow on a column containing pre-equilibrated CM Sepharose resin (GE HealthCare). The resin was washed with Sepharose wash buffer 100 mM NaCl (50 mM Tris-HCl (pH 7.5), 5 mM β-mercaptoethanol, 1 mM PMSF, 10% glycerol and 100 mM NaCl). SMARCAL1 variants were eluted with Sepharose buffer 300 mM NaCl (50 mM Tris-HCl (pH 7.5), 5 mM β-mercaptoethanol, 1 mM PMSF, 10% glycerol and 300 mM NaCl). Fractions containing high protein concentration were pooled, aliquoted, snap-frozen in liquid nitrogen and stored at −80 °C.

#### Human FANCM

FANCM was expressed in *S.* *frugiperda* 9 (*Sf*9) cells using the Bac-to-Bac expression system (Thermo Fisher Scientific), according to the manufacturer’s recommendations. The protein sequence was codon optimized for the expression in *Sf*9 insect cells (Twist Bioscience) and cloned into NheI and AflII restriction sites of pFastBac1, generating pFB-2xMBP-hFANCM-10xHis. Frozen *Sf*9 pellet from 1.5-l culture was resuspended in LB (50 mM Tris-HCl (pH 7.5), 5 mM β-mercaptoethanol, 2 mM PMSF, 1 mM EDTA, protease inhibitor cocktail (P8340; Sigma-Aldrich) diluted 1:200 and 50 µg ml^−1^ of leupeptin (Merck Millipore)) and incubated at 4 °C for 5 min. Glycerol was added to a final concentration of 16.7%. NaCl was added to a final concentration of 305 mM. The solution was incubated at 4 °C for 30 min. The mixture was centrifuged at 20,000*g* at 4 °C for 30 min. The resulting soluble extract was incubated with 24-ml amylose resin (NEB) at 4 °C for 1 h. The resin was then washed with 1 M amylose wash buffer (50 mM Tris-HCl (pH 7.5), 5 mM β-mercaptoethanol, 1 mM PMSF, 10% glycerol, 1 mM EDTA and 1 M NaCl) followed by 300 mM amylose wash buffer (50 mM Tris-HCl (pH 7.5), 5 mM β-mercaptoethanol, 1 mM PMSF, 10% glycerol and 300 mM NaCl) and 150 mM amylose wash buffer (50 mM Tris-HCl (pH 7.5), 5 mM β-mercaptoethanol, 1 mM PMSF, 10% glycerol and 150 mM NaCl). Proteins were eluted using amylose elution buffer (50 mM Tris-HCl (pH 7.5), 5 mM β-mercaptoethanol, 1 mM PMSF, 10% glycerol, 150 mM NaCl and 10 mM maltose). The MBP-tagged variants were incubated with PreScission Protease (approximately 20 μg of PreScission Protease per 100 μg of tagged protein) at 4 °C for 1 h to cleave the MBP tag. Subsequently, the cleaved protein was diluted to reduce the concentration of NaCl to approximately 100 mM with a buffer containing no NaCl (50 mM Tris-HCl (pH 7.5), 5 mM β-mercaptoethanol, 1 mM PMSF and 10% glycerol) and applied in flow on a column containing pre-equilibrated HiTrap Q HP column on the ÄKTA purifier (Cytiva). The protein was washed with 20 ml of ÄKTA buffer (50 mM Tris-HCl (pH 7.5), 5 mM β-mercaptoethanol, 1 mM PMSF and 10% glycerol) at 100 mM NaCl and eluted from the column with a salt gradient in ÄKTA buffer from 100 mM to 1 M NaCl. Fractions containing the protein were pooled, aliquoted and snap-frozen.

### Secondary structure unfolding assays

Secondary structure unfolding assays (15 μl) were carried out in a reaction buffer containing 25 mM of Tris-acetate (pH 7.5), 2 mM ATP, 2 mM magnesium acetate, 1 mM dithiothreitol, 0.1 mg ml^−1^ Recombinant albumin (NEB) and 100 ng of DNA substrate per reaction (pUC19, pUC19_TA rich or pUC19_Chr 3). The final NaCl concentration was adjusted to 50 mM accounting for the salt brought into the reactions with protein storage or protein dilution buffer. The reactions were assembled and supplemented with the relevant proteins (with and without 20 nM RPA, as indicated) on ice and then incubated at 37 °C for 30 min. The reactions were then incubated with T7 Endonuclease I (NEB) or EcoRI (NEB) for 10 min at 37 °C, and where indicated, followed by SspI (NEB) for 30 min at 37 °C. The reactions were terminated by adding 1-μl Proteinase K (14–22 mg ml^−1^; Roche) and 5-μl 0.2% stop solution (150 mM EDTA, 0.2% SDS, 30% glycerol and bromophenol blue) and incubated at 37 °C for 10 min. The products were separated by electrophoresis in 1% native agarose gels in the presence of GelRed (1:20,000; Biotium). Images of gels were acquired using Quantum (CX5 Edge; VILBER). The images were quantified using ImageJ and expressed as percentage of secondary structure removal.

### DNA substrate preparation

The pUC19_Chr3 vector was obtained by inserting a representative sequence (5′-TGTATATATATACAATATATATACATGTATATATATACATGTATATATACTGTATATATACATGTATATATATACATGTATATATACTGTATATATACAT-3′) into the SacI and XbaI restriction sites of a pUC19 vector. The selected sequence from chromosome 3 was found in ChIP–seq experiments and was predicted to be a substrate for FANCM, SMARCAL1 and MRE11. The pUC19_TA rich vector instead contains (TA)20 inserted into the KpnI and BamHI restriction sites of pUC19 (ref. ^73^).

### Liquid chromatography–tandem mass spectrometry sample preparation

Proteins were extracted in 150-μl LB (4% SDS and 50 mM Tris-HCl (pH 8.2)) using a tissue homogenizer (TissueLyser II; QIAGEN) with glass beads and 2 × 2 min cycles at 30 Hz. The samples were treated with high-intensity focused ultrasound (HIFU) for 1 min at an ultrasonic amplitude of 100% before boiling at 95 °C for 5 min while shaking at 800 rpm on a thermoshaker (Eppendorf). Cell debris and other insoluble components were separated by centrifugation at 20,000*g* for 10 min. Protein concentration was determined using the Lunatic UV/Vis polychromatic spectrophotometer (Unchained Labs).

For each sample, a volume corresponding to 780 µg of protein was taken and supplemented with 5 mM tris(2-carboxyethyl)phosphine and 15 mM chloroacetamide at 30 °C for 30 min in the dark.

Samples were processed using the single‐pot solid‐phase-enhanced sample preparation. Protein purification, digest and peptide clean-up were performed using a KingFisher Flex System (Thermo Fisher Scientific) and Carboxylate-Modified Magnetic Particles (GE65152105050250 and GE45152105050250; GE Life Sciences)^74^. Samples were diluted with 100% ethanol to a final concentration of 60% ethanol. The beads, wash solutions (80% ethanol) and samples were loaded into 96-deep-well or microplates and transferred to the KingFisher. Collection of beads, protein binding to beads, washing of beads, protein digestion (overnight at 37 °C with a trypsin:protein ratio of 1:50 in 50 mM triethylammonium bicarbonate) and peptide elution were carried out on the robotic system. The digest solution and water elution were combined and dried to completeness.

Antibody-based enrichment of the ubiquitin remnant motif (K-ε-GG) was performed according to the manufacturer’s protocol (PTMScan; Cell Signaling Technology), except for using only 40% of the recommended bead volume. Both eluates were loaded directly onto a preconditioned Evotip (Evosep Biosystems) according to the manufacturer’s protocol.

### Liquid chromatography–tandem mass spectrometry data acquisition (proteomics)

Mass spectrometry analysis of proteomics samples was performed on a timsTOF Pro (Bruker) coupled to an Evosep One (Evosep Biosystems). Samples were separated using the 15 samples per day method while keeping the analytical column (PepSep C18, 15 cm × 150 µm; 1.5 µm) at 50 °C. Mass spectrometry scans were acquired from *m*/*z* 100 to *m*/*z* 1,700 with an inverse mobility ramp (1/K0), where K0 represents the reduced ion mobility, from 0.60 to 1.60 Vs cm^−2^ (ion accumulation and ramp time both set at 100 ms). MS2 scans were acquired in data-independent acquisition parallel accumulation–serial fragmentation mode. One mass spectrometry scan was followed by 16 parallel accumulation–serial fragmentation cycles from *m*/*z* 400 to *m*/*z* 1,200, with overlapping isolation windows of *m*/*z* 26, covering 2 × 0.30 (1/K0) ion mobility windows in the range of 0.60–1.42 Vs cm^−2^. Singly charged ions were excluded using the ion mobility polygon filter mask. The mass spectrometry proteomics data were handled using the local laboratory information management system B-Fabric^75^.

### Protein identification and quantification

The acquired mass spectrometry data were processed for identification and quantification using Spectronaut (v.19.0240606.62635; Biognosys) in directDIA mode. Spectra were searched against a canonical Swiss-Prot database for human and common protein contaminants (NCBI taxonomy ID 9606; release date 30 March 2023). Carbamidomethylation of cysteine was set as fixed modification, whereas methionine oxidation, N-terminal protein acetylation, GlyGly and LeuArgGlyGly on lysine residues were set as variable modifications. Enzyme specificity was set to trypsin/P allowing a minimal peptide length of seven amino acids and a maximum of two missed cleavages. Precursor and fragment tolerance was set to dynamic for the initial search. The maximum false discovery rate was set to 0.01 for peptides and 0.01 for proteins. Protein quantification was performed in Spectronaut using the default post-translational modification settings. The quantitative data were extracted using the BGS Factory Report (default) and used for follow-up analyses.

For statistical evaluation and site-specific integration of the results, the Spectronaut modification output was filtered for only confidently assigned diGly modified peptides, and for each diGly peptide assignment, the number of diGly modifications, the modified residue and position in the protein were parsed into a data frame. Further, a new identifier was generated that comprised the protein accession, the diGly acceptor residue and the diGly site in the full-length protein. This identifier was then used to summarize (sum) quantities from each sample. This site-centric data are then further analysed using the prolfqua R package^76^.

For this, the data are log_2_-transformed and normalized with a robust *z*-score transformation. A linear model is fitted to each diGly peptide abundance to compute the significant differences between different conditions. These group comparisons (contrasts) were evaluated with a moderated Wald test with pooled variance (as implemented in the limma R package^77^. The resulting *P* values were adjusted for multiple testing using the Benjamini–Hochberg method. All relevant mass spectrometry data are included in the Source data Excel file.

### Reporting summary

Further information on research design is available in the Nature Portfolio Reporting Summary linked to this article.

## Online content

Any methods, additional references, Nature Portfolio reporting summaries, source data, extended data, supplementary information, acknowledgements, peer review information; details of author contributions and competing interests; and statements of data and code availability are available at 10.1038/s41586-025-08815-4.

## Supplementary information

Supplementary File 1 This file contains Supplementary Figs. 1 (gating strategies for flow cytometry) and 2 (uncropped gels and blots) and methods (modified GEMINI calculate LFC function).

Reporting Summary

Supplementary Table 1 LOFTEE analysis of RPE-1 cells confirms that they have no known deleterious mutations in key DDR genes.

Supplementary Table 2 All SPIDR screen library elements and sgRNA sequences.

Supplementary Table 3 SPIDR screen GEMINI scores.

Supplementary Table 4 GEMINI scores for the focused screens in K562 and HeLa S3 cells, as well as for corresponding gene pairs in the SPIDR screen.

Supplementary Table 5 Annotated ChIP-Seq peaks table.

Supplementary Tables 6 and 7 Supplementary Table 6. CRISPRi and CRISPR KO sgRNA sequences. Supplementary Table 7. Key resources and reagents.

Supplementary Table 8 Normalized sgRNA counts for the SPIDR screen.

## Source data

Source Data Figs. 1, 2, 3 and 4 and Extended Data Figs. 3, 4, 5, 6, 7, 8, 9, 10 and 12

## Data Availability

The CRISPRi screen dataset is available through the NCBI BioProject database (BioProject: PRJNA988447). ChIP–seq data are available through GEO (GSE236062). Repli-seq data for GM06990 cells are available through GEO (GSM923443). Source data are provided with this paper.

## Code availability

The modified gemini_calculate_lfc_mod function is provided in the Supplementary Information.
