# Supplementary material for: Comprehensive interrogation of synthetic lethality in the DNA damage response
Source: Nature. 2025 Apr 9;640(8060):1093–102. doi: 10.1038/s41586-025-08815-4 (PMC12018271; doi:10.1038/s41586-025-08815-4)
Supplement: Supplementary file 1 — This file contains Supplementary Figs. 1 (gating strategies for flow cytometry) and 2 (uncropped gels and blots) and methods (modified GEMINI calculate LFC function). [file 41586_2025_8815_MOESM1_ESM.docx]

Supplementary File 1

Contents

[Supplementary Figures 2](#_heading=h.gjdgxs)

[Supplementary Figure 1: Gating strategies for flow cytometry 2](#_heading=h.30j0zll)

[Supplementary Figure 2 – Uncropped blots and gel scans 3](#_heading=h.1fob9te)

[Supplementary Figure 2 (continued) – Uncropped blots and gel scans 4](#_heading=h.3znysh7)

[Supplementary Figure 2 (continued) – Uncropped blots and gel scans 5](#_heading=h.2et92p0)

[Supplementary Figure 2 (continued) – Uncropped blots and gel scans 6](#_heading=h.tyjcwt)

[Supplementary Methods – modified GEMINI calculate LFC function 10](#_heading=h.1t3h5sf)

# Supplementary Figures

## Supplementary Figure 1: Gating strategies for flow cytometry


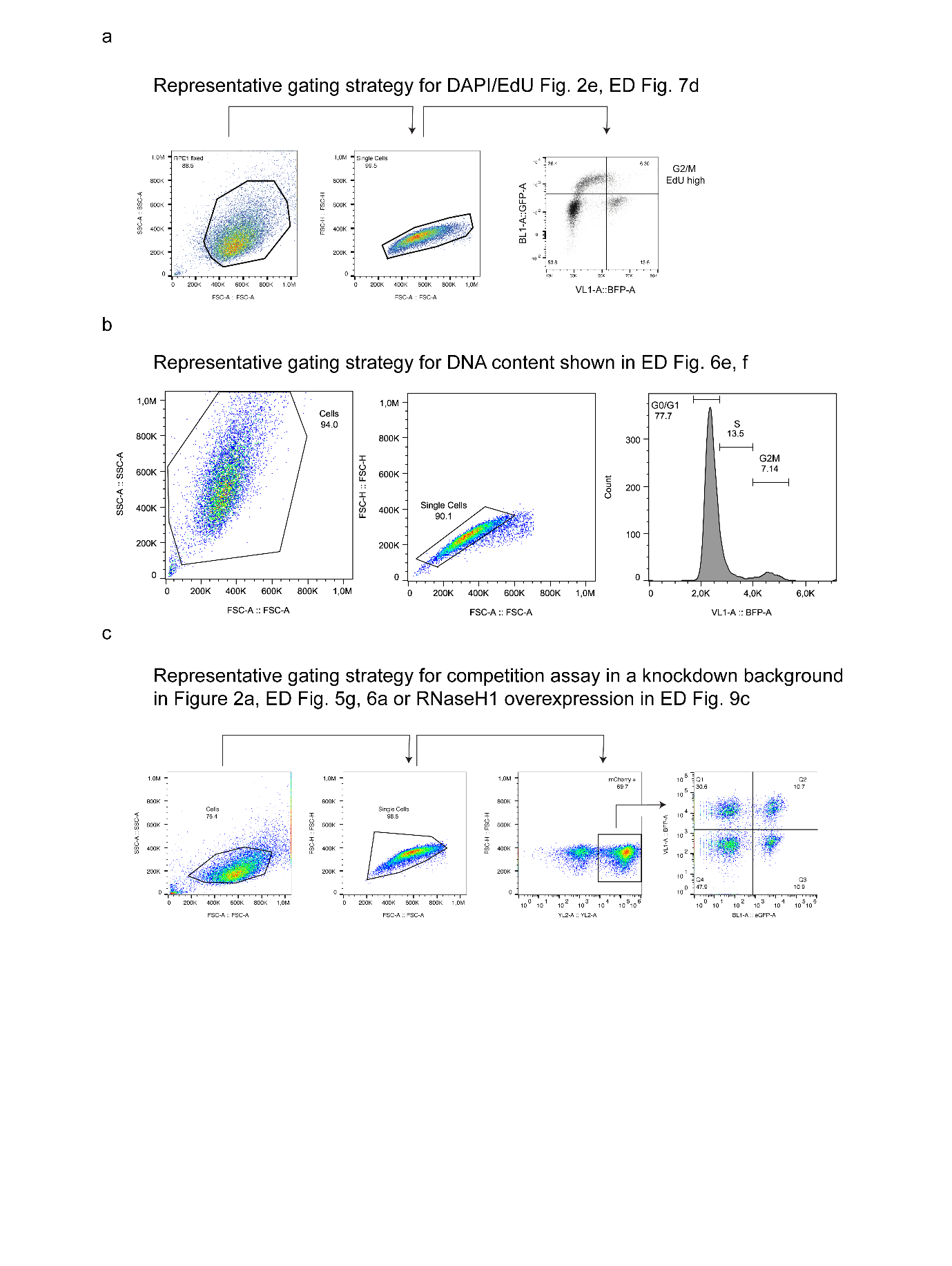


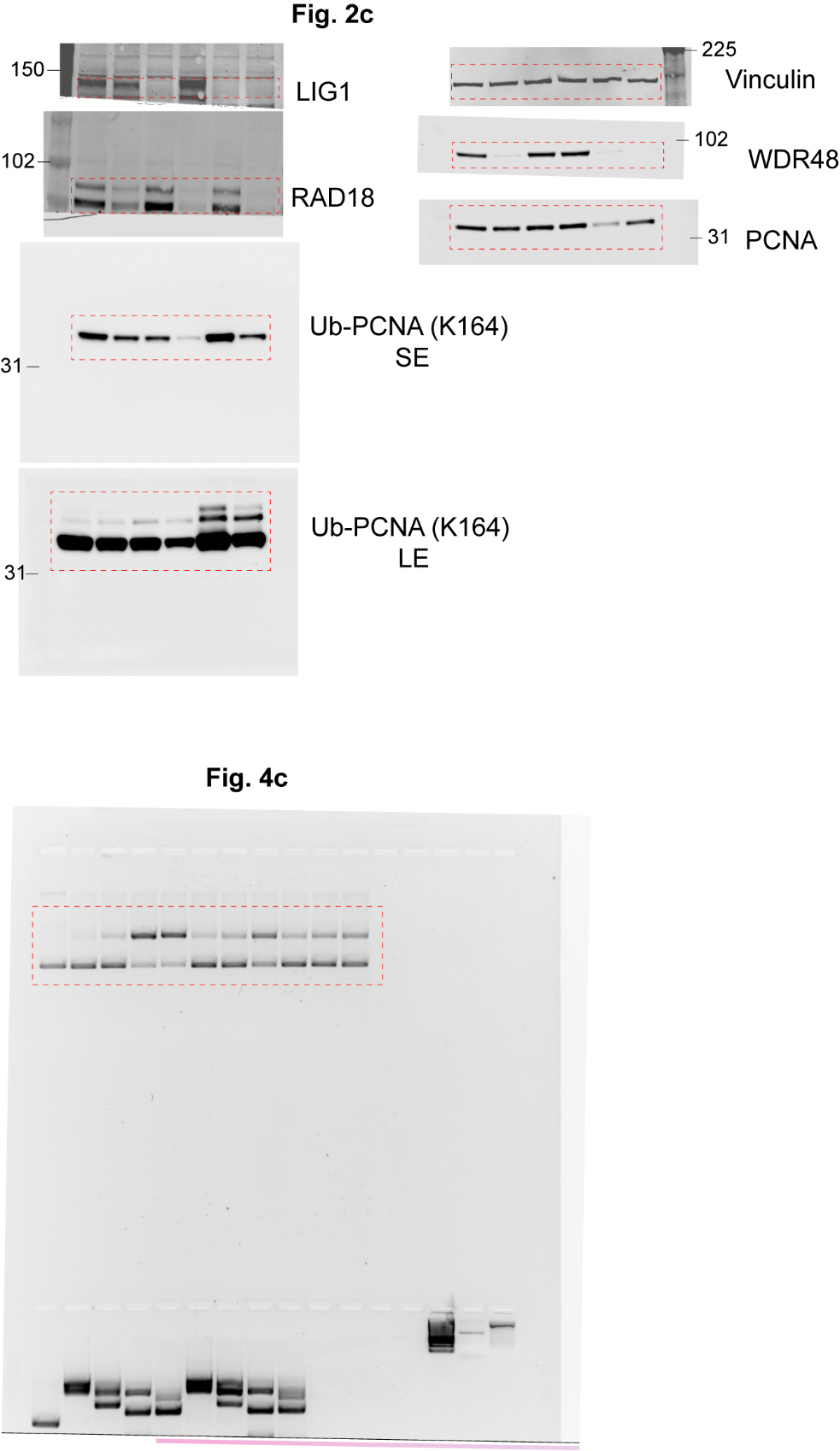


## Supplementary Figure 2 – Uncropped blots and gel scans


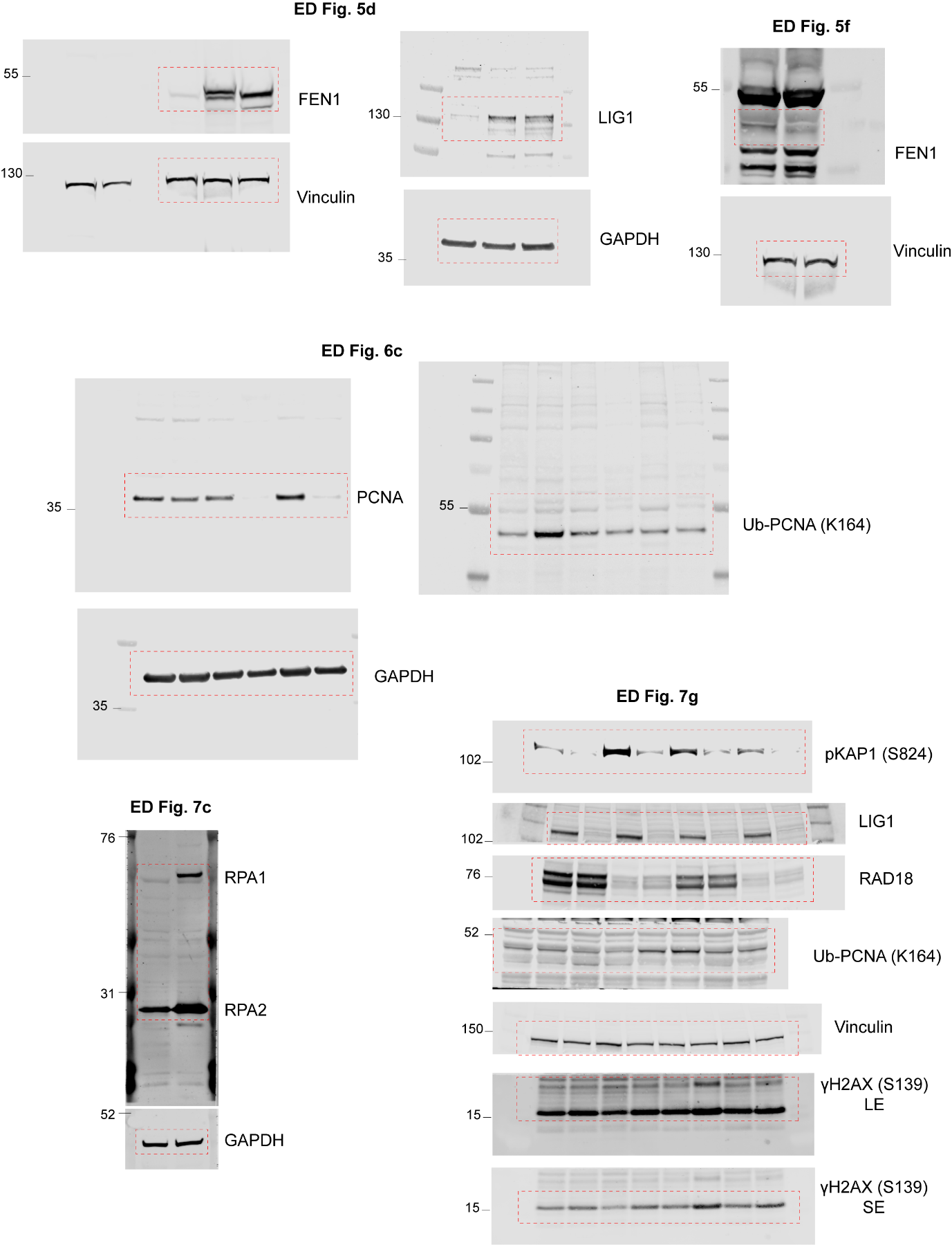


## Supplementary Figure 2 (continued) – Uncropped blots and gel scans


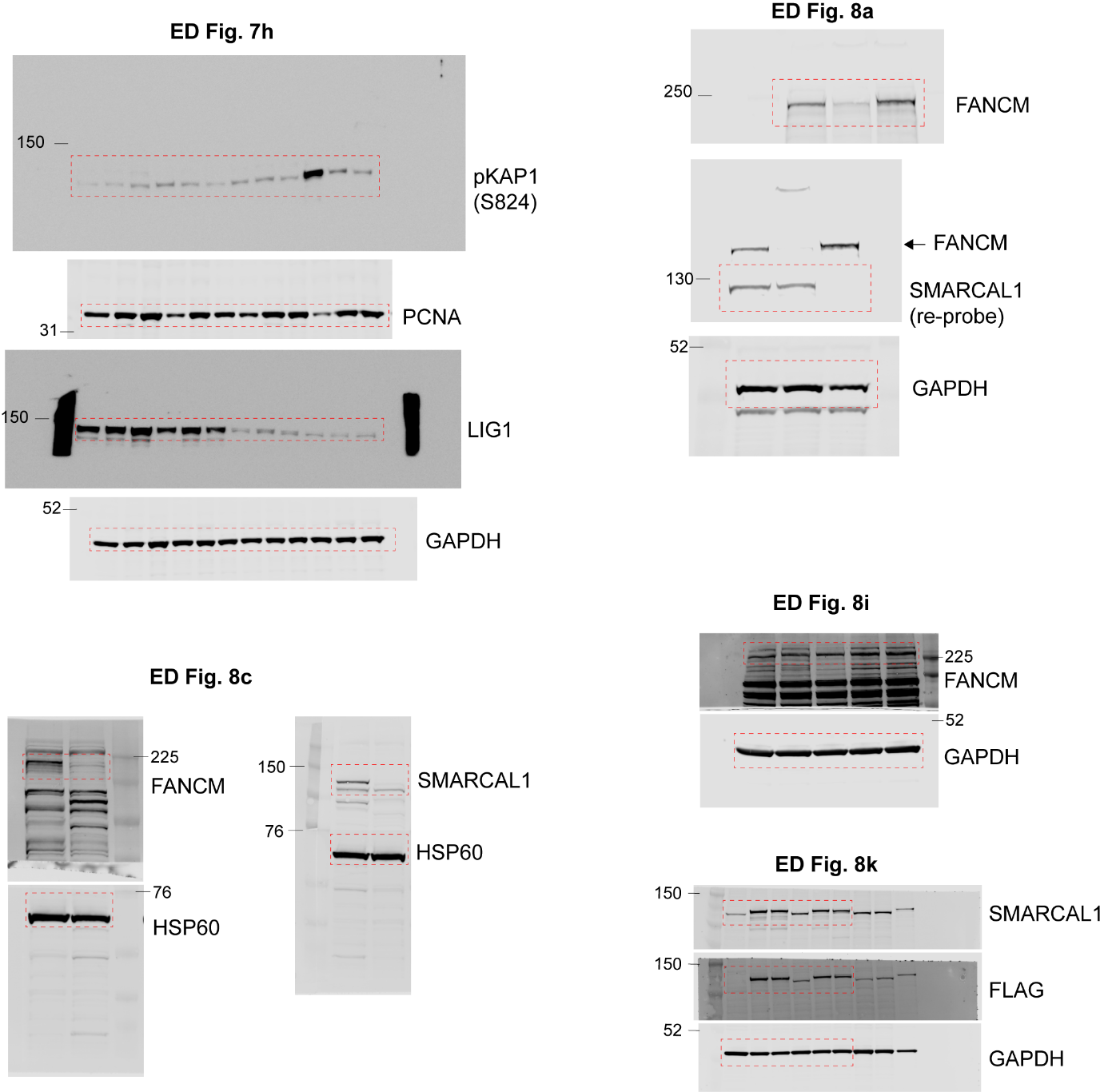


## Supplementary Figure 2 (continued) – Uncropped blots and gel scans


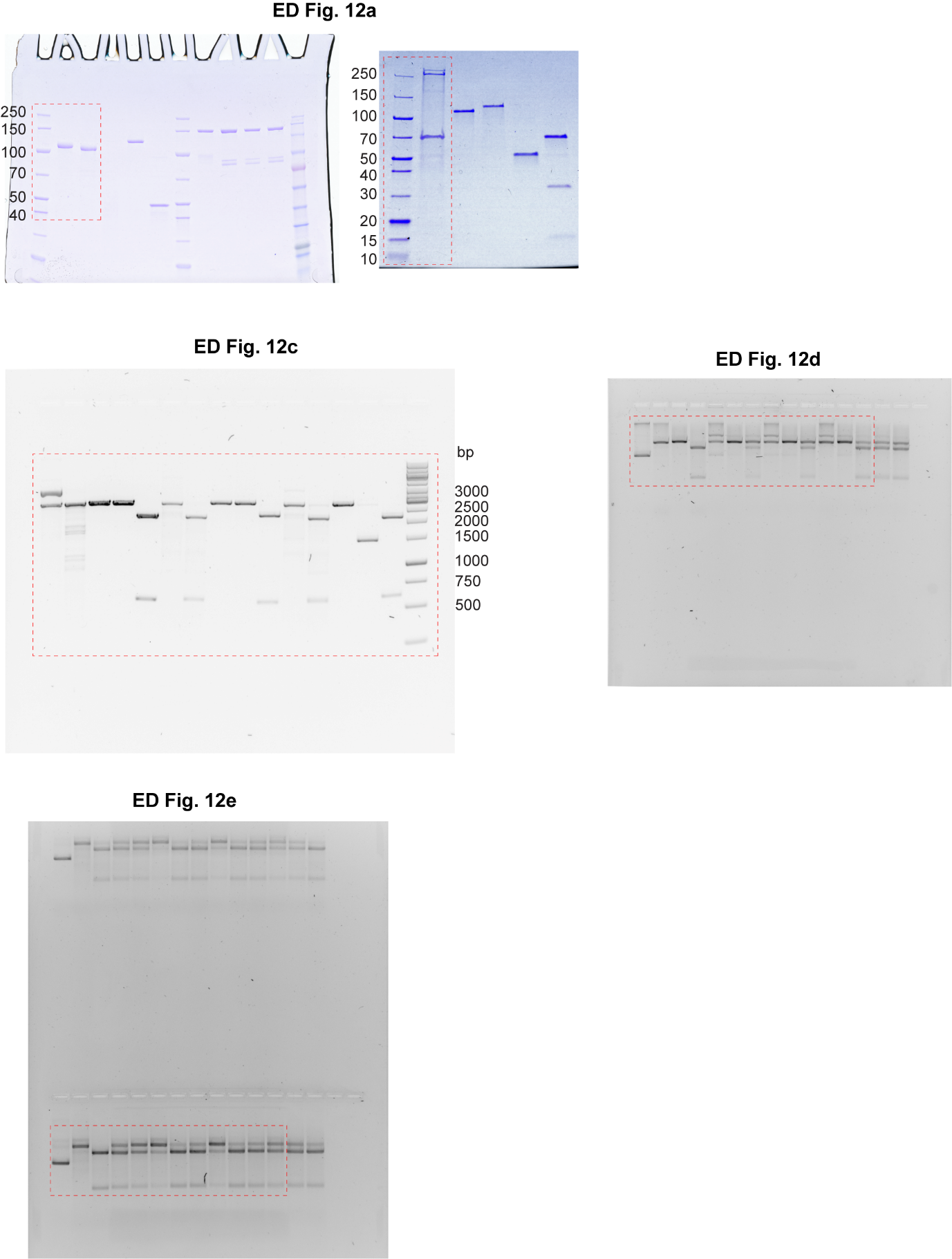


## Supplementary Figure 2 (continued) – Uncropped blots and gel scans

gemini_calculate_lfc_mod <- **function**(Input,

counts = "counts",

sample.column.name = "samplename",

normalize = TRUE,

CONSTANT = 10) {

stopifnot("gemini.input" %**in**% class(Input))

stopifnot(counts %**in**% names(Input))

*# normalize and scale counts*

mat <- Input[[counts]]

total.counts = sum(mat, na.rm = TRUE)

SCALE = (total.counts / ncol(mat))

**if** (normalize) {

norm.mat = apply(mat, 2, **function**(x) {

x = ((x / sum(x, na.rm = TRUE)) * SCALE) + CONSTANT

})

Input[[paste0("normalized_", counts)]] <- norm.mat

*# compute median-normalized log counts*

data <- norm.mat

} **else**{

data <- mat

}

data = apply(data, 2, **function**(x) {

*## Removed - stats::median(log2(x), na.rm = TRUE) from the original function and added + CONSTANT to include the pseudocount of 10 to all counts*

log2(x + CONSTANT)

})

*# compute log-fold changes*

ETP.cols <- which(Input$replicate.map$TP == "ETP")

ETP.samples <-

unique(Input$replicate.map[ETP.cols, ][[sample.column.name]])

**if** (length(ETP.cols) == 0) {

**stop**(

"No ETP samples identified. Make sure at least one ETP column is specified in Input$replicate.map$TP"

)

} **else** **if** (length(ETP.cols) == 1) {

*# If only 1 ETP column specified*

ETP = data[, ETP.cols]

} **else** **if** (length(ETP.cols) > 1 &

!any(ETP.samples %**in**% Input$replicate.map[-ETP.cols, ][[sample.column.name]])) {

*# If multiple ETP replicates belonging to only 1 sample (which is not found in LTP samples, i.e. pDNA)*

ETP = data[, ETP.cols] %>%

as.data.frame() %>%

rowMeans(na.rm = TRUE)

}

*# If ETPs match LTPs by sample, that is handled here:*

LTP.cols <- which(Input$replicate.map$TP == "LTP")

LTP <- as.matrix(data[, LTP.cols])

colnames(LTP) <- Input$replicate.map$colname[LTP.cols]

LTP_df <-

lapply(unique(Input$replicate.map[[sample.column.name]][Input$replicate.map$TP == "LTP"]), **function**(x) {

**if** (!exists('ETP')) {

*# Check if an ETP dataframe has been established*

ETP.cols = which(Input$replicate.map$TP == "ETP" &

Input$replicate.map[[sample.column.name]] == x)

**if** (!length(ETP.cols) > 0)

**stop**("No ETP specified for ", x)

ETP = data[, ETP.cols] %>%

as.data.frame() %>%

rowMeans(na.rm = TRUE) *# Create ETP df for this sample*

}

cols <-

Input$replicate.map$colname[Input$replicate.map[[sample.column.name]] ==

x & Input$replicate.map$TP == "LTP"]

LFC <- LTP[, match(cols, colnames(LTP), nomatch = 0)] %>%

as.data.frame(optional = TRUE) %>%

rowMeans(na.rm = TRUE) %>%

magrittr::subtract(ETP)

**return**(LFC)

}) %>%

magrittr::set_names(unique(Input$replicate.map[[sample.column.name]][Input$replicate.map$TP == "LTP"])) %>%

dplyr::bind_cols() %>%

as.data.frame(optional = TRUE, stringsAsFactors = FALSE) %>%

magrittr::set_rownames(Input$guide.pair.annot[, 1])

*# Consolidate LFC to sample level*

unique.to.sample <-

names(which(

apply(Input$replicate.map, 2, **function**(x)

length(unique(x))) == length(unique(Input$replicate.map[[sample.column.name]]))

))

Input$sample.annot <- Input$replicate.map %>%

dplyr::filter(.$`TP` == "LTP") %>%

dplyr::select(dplyr::all_of(c(sample.column.name, 'TP', unique.to.sample))) %>%

unique() %>%

magrittr::set_rownames(seq(from = 1, to = nrow(.))) %>%

dplyr::mutate(rowname = colnames(LTP_df))

Input[["LFC"]] <- LTP_df %>%

dplyr::select(unique(Input$replicate.map[[sample.column.name]][Input$replicate.map$TP != "ETP"])) %>%

as.matrix()

*# Return object*

Output <- Input

class(Output) <- c(class(Output), "gemini.input")

**return**(Output)

}

## Supplementary Methods – modified GEMINI calculate LFC function
