# Supplementary material for: Comprehensive interrogation of synthetic lethality in the DNA damage response
Source: Nature. 2025 Apr 9;640(8060):1093–102. doi: 10.1038/s41586-025-08815-4 (PMC12018271; doi:10.1038/s41586-025-08815-4)
Supplement: Supplementary file 2 — Reporting Summary [file 41586_2025_8815_MOESM2_ESM.pdf]

Corresponding author(s): Jacob E Corn

Last updated by author(s): Feb 7, 2025

Reporting Summary

Nature Portfolio wishes to improve the reproducibility of the work that we publish. This form provides structure for consistency and transparency in reporting. For further information on Nature Portfolio policies, see our Editorial Policies and the Editorial Policy Checklist.

Statistics

For all statistical analyses, confirm that the following items are present in the figure legend, table legend, main text, or Methods section.

n/a	Confirmed	
☐	☒	The exact sample size (*n*) for each experimental group/condition, given as a discrete number and unit of measurement
☐	☒	A statement on whether measurements were taken from distinct samples or whether the same sample was measured repeatedly
☐	☒	The statistical test(s) used AND whether they are one- or two-sided<br>*Only common tests should be described solely by name; describe more complex techniques in the Methods section.*
☒	☐	A description of all covariates tested
☐	☒	A description of any assumptions or corrections, such as tests of normality and adjustment for multiple comparisons
☐	☒	A full description of the statistical parameters including central tendency (e.g. means) or other basic estimates (e.g. regression coefficient) AND variation (e.g. standard deviation) or associated estimates of uncertainty (e.g. confidence intervals)
☐	☒	For null hypothesis testing, the test statistic (e.g. *F*, *t*, *r*) with confidence intervals, effect sizes, degrees of freedom and *P* value noted<br>*Give P values as exact values whenever suitable.*
☒	☐	For Bayesian analysis, information on the choice of priors and Markov chain Monte Carlo settings
☒	☐	For hierarchical and complex designs, identification of the appropriate level for tests and full reporting of outcomes
☒	☐	Estimates of effect sizes (e.g. Cohen's *d*, Pearson's *r*), indicating how they were calculated

Our web collection on statistics for biologists contains articles on many of the points above.

Software and code

Policy information about availability of computer code

Data collection	Flow cytometry: Data was collected using an Attune NxT Flow Cytometer (Invitrogen)<br>Microscopy: Images were acquired using an Leica SP8 confocal microscope or ZEISS Apotome 3<br>Western blots: membranes were scanned using a Li-Cor Near-InfraRed fluorescence Odyssey CLx Imaging System<br>Sequencing: paired-end sequencing was performed on a NovaSeq 6000 system (Illumina). Single-end read sequencing was performed on a NextSeq 2000 device (Illumina).<br>(RT-)qPCR: Data was acquired using a QuantStudio 6 Flex Real-Time PCR System (Applied Biosystems).
Data analysis	Flow Cytometry Analysis: Flowjo (v10.8.1)<br>Image analysis: ImageJ/FIJI (v2.9.0)<br>Data compiling, processing and statistical analyses: Microsoft excel (v2306), R Studio (2023.03.1+446 "Cherry Blossom" Release), R (v4.1.2), GraphPad Prism 9 (v9.5.0).<br>Screen Analysis: BBMap, GEMINI (https://github.com/sellerslab/gemini). Modified code is provided as a separate supplementary information html file.<br>ChIP-Seq: bowtie2 (v2.4.4), samtools (v1.6), Macs3 (v3.0.0b1), ENCODE blacklist (v2), bamCoverage (v3.3.0), CrossMap (v0.6.0), MEME-ChIP (v5.5.1)<br>Repli-Seq: Deeptools (v3.5.1)

For manuscripts utilizing custom algorithms or software that are central to the research but not yet described in published literature, software must be made available to editors and reviewers. We strongly encourage code deposition in a community repository (e.g. GitHub). See the Nature Portfolio guidelines for submitting code & software for further information.

Data

Policy information about availability of data

All manuscripts must include a data availability statement. This statement should provide the following information, where applicable:

- Accession codes, unique identifiers, or web links for publicly available datasets
- A description of any restrictions on data availability
- For clinical datasets or third party data, please ensure that the statement adheres to our policy

ChIP-Seq data: available through GEO (accession code: GSE236062).
CRISPR screen data: available through the NCBI BioProject database (BioProject: PRJNA988447).

Research involving human participants, their data, or biological material

Policy information about studies with human participants or human data. See also policy information about sex, gender (identity/presentation), and sexual orientation and race, ethnicity and racism.

Reporting on sex and gender	n/a
Reporting on race, ethnicity, or other socially relevant groupings	n/a
Population characteristics	n/a
Recruitment	n/a
Ethics oversight	n/a

Note that full information on the approval of the study protocol must also be provided in the manuscript.

Field-specific reporting

Please select the one below that is the best fit for your research. If you are not sure, read the appropriate sections before making your selection.

☒ Life sciences ☐ Behavioural & social sciences ☐ Ecological, evolutionary & environmental sciences

For a reference copy of the document with all sections, see nature.com/documents/nr-reporting-summary-flat.pdf

Life sciences study design

All studies must disclose on these points even when the disclosure is negative.

Sample size	No statistical methods were used to predetermine sample size.<br>Sample sizes were based on common practice in the field and own experience.<br>Sample sizes are indicated in the corresponding figure legends.
Data exclusions	Dual-color flow cytometry assays were halted if initial lentiviral transductions were inefficient. No data were excluded from analyses.
Replication	All attempts at replication were successful<br>The number of experimental replicates are indicated in the corresponding figure legends.
Randomization	No randomization was carried out for experiments described in this study.<br>Experiments were performed with cell lines and recombinant proteins. No assignments into experimental groups was performed.
Blinding	Analysis of 53BP1 and BrdU/RPA32 foci counts was carried out in a blinded manner using the ImageJ/Fiji Blind Analysis Tools.<br>In most other cases, measurements were performed using automated devices (e.g. flow cytometers, gel scanners, mass spectrometry, or NGS). Therefore no blinding was required.<br>Investigators were otherwise not blinded during data collection or data analysis. Differences in morphology and cell responses to drug treatments (e.g. USP1 inhibition) or depletions were highly indicative of the sample identity, making blinding unfeasible.

Reporting for specific materials, systems and methods

We require information from authors about some types of materials, experimental systems and methods used in many studies. Here, indicate whether each material, system or method listed is relevant to your study. If you are not sure if a list item applies to your research, read the appropriate section before selecting a response.

Materials & experimental systems

n/a	Involved in the study
☐	☒ Antibodies
☐	☒ Eukaryotic cell lines
☒	☐ Palaeontology and archaeology
☒	☐ Animals and other organisms
☒	☐ Clinical data
☒	☐ Dual use research of concern
☒	☐ Plants

Methods

n/a	Involved in the study
☐	☒ ChIP-seq
☐	☒ Flow cytometry
☒	☐ MRI-based neuroimaging

Antibodies

Antibodies used

WDR48 (Proteintech Cat# 16503-1-AP, RRID:AB_2878266)
FEN1 (Abcam Cat# ab153825, RRID:AB_2938984)
KAP1 (phospho S824) (Abcam Cat# ab70369, RRID:AB_1209417)
DNA Ligase I (Proteintech Cat# 18051-1-AP, RRID:AB_2265726)
Rad18 (D2B8) XP® (Cell Signaling Technology Cat# 9040, RRID:AB_2756446)
Ubiquityl-PCNA (Lys164) (D5C7P) (Cell Signaling Technology Cat# 13439, RRID:AB_2798219)
Phospho-Histone H2A.X (Ser139) (20E3) (Cell Signaling Technology Cat# 9718, RRID:AB_2118009)
Vinculin antibody [VIN-54] (Abcam Cat# ab130007, RRID:AB_11156698)
53BP1 (Novus Cat# NB100-304SS, RRID:AB_920462)
FANCM (Abcam Cat# ab95014, RRID:AB_10675719)
FANCM (Sigma-Aldrich Cat# SAB1407805, RRID:AB_10760703)
SMARCAL1 (E-12) (Santa Cruz Biotechnology Cat# sc-376377, RRID:AB_10987841)
SMARCAL1 (Thermo Fisher Scientific Cat# PA5-54181, RRID:AB_2647610)
HSP60 (N-20) (Santa Cruz Biotechnology Cat# sc-1052, RRID:AB_631683)
GAPDH, Clone D4C6R (Cell Signaling Technology Cat# 97166, RRID:AB_2756824)
FLAG (Sigma-Aldrich Cat# F3165, RRID:AB_259529)
MRE11 (Novus Cat# NB100-142, RRID:AB_10077796)
CD55 (BioLegend Cat# 311312, RRID:AB_2075856)
CldU/BrdU (Abcam Cat# ab6326, RRID:AB_305426)
IdU/BrdU (BD Biosciences Cat# 347580, RRID:AB_10015219)
RPA32 (Abcam Cat# ab10359, RRID:AB_297095)
RPA70 (RPA70 Cell Signaling Technology,Cat# 2267, RRID: AB_2180506)
Cruciform DNA (MediMabs Cat# MM-0027, RRID:AB_1808132)
Goat anti-Mouse IgG Alexa Fluor™ 488 (Thermo Fisher Scientific Cat# A-11001, RRID:AB_2534069)
Goat anti-Rabbit IgG Alexa Fluor™ 488 (Thermo Fisher Scientific # A-11008, RRID:AB_143165)
Goat anti-Rat IgG (Alexa Fluor™ 568 Molecular Probes Cat# A-11077, RRID:AB_141874)
IRDye 800CW (Donkey anti-Rabbit IgG LI-COR Biosciences Cat# 926-32213, RRID:AB_621848)
IRDye 800CW (Donkey anti-Mouse IgG LI-COR Biosciences Cat# 926-32212, RRID:AB_621847)

Validation

The specificity of the FANCM, SMARCAL1, RAD18, LIG1, FEN1, and WDR48 antibodies was evidenced by the lack of signal in samples in which their expression was either knocked out or knocked down. See ED Fig. 8a for FANCM and SMARCAL1. See Fig. 2c for LIG1, RAD18, and WDR48. See ED Fig. 5f for FEN1. The Ub-PCNA antibody was validated by a decrease in signal upon RAD18 depletion. The Phospho-Histone H2A.X (Ser139) (20E3) has been used in over 2000 publications. The 53BP1 antibody was used in over 700 publications. The KAP1 (phospho S824) and CldU/BrdU antibodies were validated by the manufacturer. The IdU/BrdU antibody has been employed in over 350 publications. The RPA32 and RPA70 antibodies were validated by an increase in signal upon their cDNA overexpression (ED Fig. 7c). The anti-cruciform DNA monoclonal antibody (2D3) has been employed in numerous studies (PMID: 15208332; PMID: 7473756; PMID: 12167636). The MRE11 antibody was extensively validated in PMID: 32313254. Loading control antibodies (vinculin, GAPDH, HSP60) are all commonly used and produced bands of the expected size by Western blot. The FANCM (Sigma-Aldrich) and SMARCAL1 (Themo Fisher Scientific) used for ChIP-Seq were validated in PMID: 32769987 and PMID: 36085347, respectively.

Eukaryotic cell lines

Policy information about cell lines and Sex and Gender in Research

Cell line source(s)

RPE-1, K562, HEK293, and HEK293T cells were obtained from either ATCC or Berkeley Cell Culture. RPE-1 p53 knockout cells were obtained from the Stephen P Jackson Lab (Cambridge). HeLa S3 cells were obtained from the Nicola Aceto lab (ETH Zurich).

Authentication

All parental cell lines were obtained authenticated (by STR profiling).

The RPE-1 p53-proficient and -deficient cell lines were re-validated by STR profiling.

Mycoplasma contamination

All cell lines regularly tested negative for mycoplasma.

Commonly misidentified lines
(See ICLAC register)

No commonly misidentified cell lines were used in this study.

Plants

Seed stocks	n/a
Novel plant genotypes	n/a
Authentication	n/a

ChIP-seq

Data deposition

☒ Confirm that both raw and final processed data have been deposited in a public database such as GEO.

☒ Confirm that you have deposited or provided access to graph files (e.g. BED files) for the called peaks.

Data access links
May remain private before publication.

https://www.ncbi.nlm.nih.gov/geo/query/acc.cgi?acc=GSE236062

Files in database submission

rep1_Double-SDel-sgFM_R1.fastq.gz
rep2_Double-SDel-sgFM_R1.fastq.gz
rep3_Double-SDel-sgFM_R1.fastq.gz
rep1_SDel_R1.fastq.gz
rep2_SDel_R1.fastq.gz
rep3_SDel_R1.fastq.gz
rep1_sgFM_R1.fastq.gz
rep2_sgFM_R1.fastq.gz
rep3_sgFM_R1.fastq.gz
rep1_RPE1_WT_R1.fastq.gz
rep2_RPE1_WT_R1.fastq.gz
rep3_RPE1_WT_R1.fastq.gz
rep1_Double-SDel-sgFM_R2.fastq.gz
rep2_Double-SDel-sgFM_R2.fastq.gz
rep1_SDel_R2.fastq.gz
rep2_SDel_R2.fastq.gz
rep1_sgFM_R2.fastq.gz
rep2_sgFM_R2.fastq.gz
rep1_RPE1_WT_R2.fastq.gz
rep2_RPE1_WT_R2.fastq.gz
FANCM_WT_R1.fastq.gz
FANCM_WT_R2.fastq.gz
FANCM_SMARCAL1KO_R1.fastq.gz
FANCM_SMARCAL1KO_R2.fastq.gz
SMARCAL1_WT_R1.fastq.gz
SMARCAL1_WT_R2.fastq.gz
SMARCAL1_FANCMKO_R1.fastq.gz
SMARCAL1_FANCMKO_R2.fastq.gz
WT_X_R1.fastq.gz
WT_X_R2.fastq.gz
SMARCAL1KO_X_R1.fastq.gz
SMARCAL1KO_X_R2.fastq.gz
sgFANCM_X_R1.fastq.gz
sgFANCM_X_R2.fastq.gz
SMARCAL1KOsgFANCM_X_R1.fastq.gz
SMARCAL1KOsgFANCM_X_R2.fastq.gz
doubles_R1.fastq.gz
doubles_R2.fastq.gz
doubles_ERCC1_R1.fastq.gz
doubles_ERCC1_R2.fastq.gz
doubles_ERCC4_R1.fastq.gz
doubles_ERCC4_R2.fastq.gz
doubles_GEN1_R1.fastq.gz
doubles_GEN1_R2.fastq.gz
doubles_MUS81_R1.fastq.gz
doubles_MUS81_R2.fastq.gz

Genome browser session
(e.g. UCSC)

http://genome-euro.ucsc.edu/s/mschroeder/ChIPseqCornlabMRE11_sgFM_SDel_DoubleKO

Methodology

Replicates	MRE11 (n =3)

Replicates	FANCM, SMARCAL1, Cruciform, MRE11 plus nuclease depletions (n =1)
Sequencing depth	sample seq_depth total_reads mapped read_length single/paired<br>rep1_RPE1 4.0 244808474 237484142 51 paired-end<br>rep1_SDel 4.7 286460276 271694662 51 paired-end<br>rep1_sgFM 2.6 162239884 87970521 51 paired-end<br>rep1_double-sdel-sgfm 4.0 247484896 237941902 51 paired-end<br>rep2_RPE1 4.0 247234062 49347248 51 paired-end<br>rep2_SDel 3.4 206608462 38703256 51 paired-end<br>rep2_sgFM 4.0 247550260 29144752 51 paired-end<br>rep2_double-sdel-sgfm 3.3 201773938 22682742 51 paired-end<br>rep3_RPE1 7.5 117834233 110406866 201 single-end<br>rep3_SDel 5.9 92688711 86994099 201 single-end<br>rep3_sgFM 5.1 78879042 67270898 201 single-end<br>rep3_double-sdel-sgfm 5.0 77764029 63861742 201 single-end<br>SMARCAL1KO_X 0.54 11171784 10898738 151 paired-end<br>SMARCAL1KOsgFANCM_X 1.55 32173244 31441866 151 paired-end<br>WT_X 0.10 2050318 1867962 151 paired-end<br>sgFANCM_X 1.80 37425284 36916915 151 paired-end<br>doubles_ERCC1 1.70 35400324 24772709 151 paired-end<br>doubles_ERCC4 1.19 24771516 21149660 151 paired-end<br>doubles_GEN1 1.30 26938814 15669040 151 paired-end<br>doubles_MUS81 1.86 38586508 21160019 151 paired-end<br>doubles 0.43 8867848 5748817 151 paired-end<br>FANCM_SMARCAL1KO 0.63 38675926 31541080 51 paired-end<br>FANCM_WT 0.67 41402374 35603931 51 paired-end<br>SMARCAL1_FANCMKO 0.89 18473936 18095368 151 paired-end<br>SMARCAL1_WT 0.81 16749226 16536513 151 paired-end
Antibodies	MRE11 (Cat# NB100-142, Novus Biologicals), FANCM Sigma-Aldrich (Cat# SAB1407805, RRID:AB_10760703), SMARCAL1 (Thermo Fisher Scientific Cat# PA5-54181, RRID:AB_2647610), Cruciform (DNA MediMabs Cat# MM-0027, RRID:AB_1808132)
Peak calling parameters	For single-end data: bowtie2 --local --fast-local -N 0 -k 1 -q --threads 90 -x ref-genome -U sample.fastq.gz | samtools view -bS -@ 10 - | samtools sort -@ 20 - > sample.bam<br><br>for paired-end: bowtie2 --local --fast-local -N 0 -k 1 -q --threads 90 -x ref-genome -1 sample_R1.fastq.gz -2 sample_R2.fastq.gz | samtools view -bS -@ 10 - | samtools sort -@ 20 - > sample.bam<br><br>for macs3 data: macs3 callpeak -t sample.bam -c RPE1_WT.bam --broad -f BAM -g hs -n sample -B -q 0.01
Data quality	Peaks were filtered using the ENCODE blacklist v2
Software	Raw paired-end FASTQ files were aligned to the GRCh38 reference genome using bowtie2 version 2.4.4.

Flow Cytometry

Plots

Confirm that:

☒ The axis labels state the marker and fluorochrome used (e.g. CD4-FITC).

☒ The axis scales are clearly visible. Include numbers along axes only for bottom left plot of group (a 'group' is an analysis of identical markers).

☒ All plots are contour plots with outliers or pseudocolor plots.

☒ A numerical value for number of cells or percentage (with statistics) is provided.

Methodology

Sample preparation	For detection of intracellular BFP, GFP, or mCherry, cells (RPE1, HEK293, K562) were washed once in 1X PBS, and immediately analyzed.<br>For DNA content (DAPI) analysis, RPE1 cells were fixed in 70% ethanol for 30 minutes on ice, washed once 1X PBS + 5% FBS, and stained at room temperature using 1 µg/ml DAPI solution (BD Biosciences) diluted in 1X PBS + 5% FBS for 5 minutes.<br>For CD55 staining, cells were stained with primary antibody in 1X PBS (1% FBS) for 10-30 minutes at room temperature, harvested by centrifugation, washed once in 1X PBS (1% FBS), and analyzed.
Instrument	Attune NxT Flow Cytometer (with autosampler), Thermo Fisher.
Software	Data were acquired Attune NxT Software v3.2.1 and analyzed with FlowJo (v10.8.1).
Cell population abundance	For each sample, >5,000 events (gated on live cells) were collected.

Gating strategy	Cells were gated on live cells (SSC-A vs FSC-A), then single cells (FSC-H vs FSC-A). GFP-positive cells were gated on SSC-A vs BL1-A. BFP-positive cells were gated on SSC-A vs VL1-A. mCherry-positive cells were gated on SSC-A vs YL1-A.

☒ Tick this box to confirm that a figure exemplifying the gating strategy is provided in the Supplementary Information.
