# Supplementary material for: Comprehensive interrogation of synthetic lethality in the DNA damage response
Source: Nature. 2025 Apr 9;640(8060):1093–102. doi: 10.1038/s41586-025-08815-4 (PMC12018271; doi:10.1038/s41586-025-08815-4)
Supplement: Supplementary file 8 — Supplementary Table 6. CRISPRi and CRISPR KO sgRNA sequences. Supplementary Table 7. Key resources and reagents. [file 41586_2025_8815_MOESM8_ESM.docx]

**Supplementary table 6: CRISPRi and CRISPR KO guide sequences used in validation studies**

| **sgRNA** | **Protospacer sequence** |
| --- | --- |
| FEN1 | GCGGGAGCGCGGGCTTTGGA |
| TERF2 | GATGGCCGCGGGGGCCGGGA |
| SUPT16H | GGGCGGGAAGAGACTGCGAG |
| ATR | GGCCCGACGGAGCCGTGTGG |
| KDM2A | GGGTGTTGTTTCCCTCACAC |
| MEN1 | GTGCGCCGCGGTGCCTAGTG |
| C19orf40/FAAP24 | GAGGCCCGAATACAGCCGGC |
| SMARCAL1_1 | GGTCTTCCGAGTGGACGGTT |
| SMARCAL1_2 | GGAGTGGACGGTTAGGCCAC |
| LIG1 | GCGCGAACTTGGGACTGCAG |
| XRCC3 | GCGGCGGGCCTCACCTCCCG |
| FANCM_1 | GAGCAGCAGCTCAACCGCTA |
| FANCM_2 | GGAAGGAAACCGATGGGGAT |
| WDR48 | GGCGGAGGAAAGTGCAGGTA |
| TRRAP | GCGGGACGGCGACCAAAGGA |
| TOP3A | GACTGGATTCGGTGCTGAGG |
| FANCD2 | GGAAAGTCGAAAACTACGGG |
| USP1 | GCCAGGAGCGCAACAAGCAG |
| USP12 | GGCCGGGAGTGCCTGAGCGC |
| USP46 | GACTGCCCATGGTGGCGCGC |
| RAD18 | GGCATCCTCGGGAGCGACCA |
| ZRANB3 | GACCTCAGGCTCCAACTCGT |
| HLTF | GTCAGGAGCGCACGACTGAA |
| FANCA | GGCCTTGGCGCCTACAGCCC |
| RFWD3 | GCGTAGGTGCATTCGGAGTG |
| FANCL | GTCCGGACTTCGAGCCATGG |
| ERCC1 | GGACTGCAGAGGGATCGAGG |
| ERCC4 | GCGACCCGGAAGAGCTTCCA |
| SLX4 | GCCGCGGCCGCGCCGGAGGT |
| MUS81 | GACGGGGCCCCAAACACAGG |
| GEN1 | GATCAGTGGGAGGACCCGGG |
| CHEK1 perfect | GGGCAAAGGACAGTCCGGTG |
| CHEK1 mismatch | GGGCAAAGGACAGTCCGGGG |
| RPA1 perfect | GGGAAGCTGGAGCTGTTGCG |
| RPA1 mismatch | GGGAAGCTGGAGTTGTTGCG |
| RAD9A perfect | GGAAGGGACCCCGGACCCGG |
| RAD9A mismatch | GGAAGGGACCCCGGACCTGG |
| BRCA1 perfect | GCACAGCCTGTCCCCCGTCC |
| BRCA1 mismatch | GCACAGCCGGTCCCCCGTCC |
| CTRL (non-targeting; pLG1-mCherry) | GATATCCCGGTGGGGTTCTC |
| CTRL (non-targeting; pSLQ-1371-BFP) | GTCAGTGCGGCGGGGACGAA |
| FANCM_KO | GAAATTGTACATGACCACGG |
| SMARCAL1_KO | GGAGAAGGTGAGGCCCATAT |

**Supplementary table 7: Key resources & reagents**

| **Reagent or resource** | **Source** | **Identifier** |
| --- | --- | --- |
| **Antibodies** |  |  |
| WDR48 | Proteintech | Cat# 16503-1-AP, RRID:AB_2878266 |
| FEN1 | Abcam | Cat# ab153825, RRID:AB_2938984 |
| KAP1 (phospho S824) | Abcam | Cat# ab70369, RRID:AB_1209417 |
| DNA Ligase I | Proteintech | Cat# 18051-1-AP, RRID:AB_2265726 |
| Rad18 (D2B8) XP® | Cell Signaling Technology | Cat# 9040, RRID:AB_2756446 |
| Ubiquityl-PCNA (Lys164) (D5C7P) | Cell Signaling Technology | Cat# 13439, RRID:AB_2798219 |
| Phospho-Histone H2A.X (Ser139) (20E3) | Cell Signaling Technology | Cat# 9718, RRID:AB_2118009 |
| Vinculin antibody [VIN-54] | Abcam | Cat# ab130007, RRID:AB_11156698 |
| 53BP1 | Novus | Cat# NB100-304SS, RRID:AB_920462 |
| 53BP1 | Thermo Fisher Scientific | Cat# A300-272A, RRID:AB_185520 |
| FANCM | Abcam | Cat# ab95014, RRID:AB_10675719 |
| FANCM | Sigma-Aldrich | Cat# SAB1407805, RRID:AB_10760703 |
| SMARCAL1 (E-12) | Santa Cruz Biotechnology | Cat# sc-376377, RRID:AB_10987841 |
| SMARCAL1 | Thermo Fisher Scientific | Cat# PA5-54181, RRID:AB_2647610 |
| HSP60 (N-20) | Santa Cruz Biotechnology | Cat# sc-1052, RRID:AB_631683 |
| GAPDH, Clone D4C6R | Cell Signaling Technology | Cat# 97166, RRID:AB_2756824 |
| FLAG | Sigma-Aldrich | Cat# F3165, RRID:AB_259529 |
| MRE11 | Novus | Cat# NB100-142, RRID:AB_10077796 |
| Cyclin A | Santa Cruz Biotechnology | Cat# sc-271682, RRID:AB_10709300 |
| RPA32 | Abcam | Cat# ab10359, RRID:AB_297095 |
| RPA70 | Cell Signaling Technology | Cat# 2267, RRID: AB_2180506 |
| CD55 | BioLegend | Cat# 311312, RRID:AB_2075856 |
| Cruciform DNA | MediMabs | Cat# MM-0027, RRID:AB_1808132 |
| CldU/BrdU | Abcam | Cat# ab6326, RRID:AB_305426 |
| IdU/BrdU | BD Biosciences | Cat# 347580, RRID:AB_10015219 |
| Goat anti-Mouse IgG Alexa Fluor™ 488 | Thermo Fisher Scientific | Cat# A-11001, RRID:AB_2534069 |
| Goat anti-Rabbit IgG  Alexa Fluor™ 488 | Thermo Fisher Scientific | # A-11008, RRID:AB_143165 |
| Goat anti-Rat IgG  Alexa Fluor™ 568 | Molecular Probes | Cat# A-11077, RRID:AB_141874 |
| IRDye 800CW Donkey anti-Rabbit IgG | LI-COR Biosciences | Cat# 926-32213, RRID:AB_621848 |
| IRDye 800CW Donkey anti-Mouse IgG | LI-COR Biosciences | Cat# 926-32212, RRID:AB_621847 |
| Anti-rabbit IgG, HRP-linked Antibody | Cell Signaling Technology | Cat#7074, RRID:AB_2099233 |
| **Chemicals, Peptides, and Recombinant Proteins** |  |  |
| FEN1-IN-1 | MedChem Express | Cat# HY-123834 |
| KSQ-4279 | MedChem Express | Cat# HY-145471 |
| Hydroxyurea | Sigma-Aldrich | Cat# H8627 |
| Mitomycin C | Sigma-Aldrich | Cat# M0503 |
| Etoposide | Sigma-Aldrich | Cat# E1383 |
| Camptothecin | Sigma-Aldrich | Cat# C9911 |
| Aphidicolin | Sigma-Aldrich | Cat# A0781 |
| Formaldehyde | Thermo Fisher Scientific | Cat# 28908 |
| Olaparib | Selleck Chemicals | Cat# S1060 |
| Pyridostatin Trihydrochloride | RayBiotech | Cat# 332-11923 |
| TMPyP4 | Sigma-Aldrich | Cat# 613560 |
| JH-RE-06 | Sigma-Aldrich | Cat# SML2993 |
| ML-323 | MedChem Express | Cat# HY-17543 |
| CldU (5-chloro-2'-deoxyuridine) | MedChem Express | Cat# CS-0059203 |
| IdU (5-iodo-2′-deoxyuridine) | Astatech Inc | Cat# AST-34454 |
| EdU (5-ethynyl-2′-deoxyuridine) | Thermo Fisher Scientific | Cat# A10044 |
| BrdU (5-Bromo-2´-Deoxyuridine) | Thermo Fisher Scientific | Cat# B23151 |
| G418 | Thermo Fisher Scientific | Cat# 11811031 |
| Puromycin Dihydrochloride | Thermo Fisher Scientific | Cat# A1113803 |
| Penicillin-Streptomycin | Thermo Fisher Scientific | Cat# 15-140-122 |
| Fetal Bovine Serum | Gibco | Cat# 10270106 |
| DMEM/F-12, GlutaMAX™ supplement | Thermo Fisher Scientific | Cat# 10565018 |
| cOmplete™, EDTA-free Protease Inhibitor Cocktail | MilliporeSigma | Cat# 11873580001 |
| Halt™ Phosphatase Inhibitor Cocktail | Thermo Fisher Scientific | Cat# 78426 |
| Polybrene | Sigma-Aldrich | Cat# H9268 |
| Trypsin EDTA (0.25%), Phenol red | Thermo Fisher Scientific | Cat# 25-200-056 |
| ProLong™ Gold Antifade Mountant with DAPI | Thermo Fisher Scientific | Cat# P36931 |
| ProLong™ Diamond Antifade Mountant | Thermo Fisher Scientific | Cat# P36965 |
| Colcemid | AdipoGen | Cat# AGCR13567M005 |
| Phosphate Buffer Saline (PBS) | Gibco | Cat# 10010023 |
| S1 nuclease | Thermo Fisher Scientific | Cat# EN0321 |
| DAPI solution | BD Biosciences | Cat# 564907 |
| Blasticidin S | Sigma-Aldrich | Cat# SBR00022 |
| Hoechst 33342 Ready Flow™ Reagent | Thermo Fisher Scientific | Cat# R37165 |
| SsoAdvanced Universal SYBR® Green Supermix | Bio-Rad | Cat# 1725270 |
| iScript™ Reverse Transcription Supermix | Bio-Rad | Cat# 1708840 |
| **Critical Commercial Assays** |  |  |
| Gentra Puregene Cell Kit | QIAGEN | Cat# 158767 |
| NEBNext Ultra II Q5 Master Mix | New England Biolabs | Cat# M0544 |
| NEB Hi-Fi Assembly Mastermix | New England Biolabs | Cat# E2621 |
| P3 Primary Cell 4D-Nucleofector® X Kit L | Lonza | Cat# V4XP-3024 |
| RNeasy Kit | QIAGEN | Cat# 74104 |
| Click-iT™ Plus EdU Alexa Fluor™ 488 Flow Cytometry Assay Kit | Thermo Fisher Scientific | Cat# C10632 |
| PTMScan® HS Ubiquitin/SUMO Remnant Motif (K-ε-GG) Kit | Cell Signaling Technology | Cat# 59322 |
| **Experimental Models: Cell Lines** |  |  |
| hTERT RPE1 dCas9-KRAB TP53−/− | This study | N/A |
| hTERT RPE1 dCas9-KRAB TP53+/+ | This study | N/A |
| K562 dCas9-KRAB | Richardson et al., 2018 | N/A |
| HeLa S3 dCas9-ZIM3 | This study | N/A |
| HEK293 dCas9-KRAB | This study | N/A |
| **Oligonucleotides** |  |  |
| Protospacer sequences for all CRISPR KO or CRISPRi experiments | See Table S6 | N/A |
| PCR primers for screen sequencing | Replogle et al., 2022 | oJR232:  AATGATACGGCGACCACCGAGATCTACACTCTTTCCCTACACGACGCTCTTCCGATCTgtatcccttggagaaCCAcctTGTTG  oJR233: CAAGCAGAAGACGGCATACGAGATtcgccttaGTCTCGTGGGCTCGGAGATGTGTATAAGAGACAGCTATGCTGTTTCCAGCtTAGCTCTtAAAC |
| qPCR primers |  | GAPDH fwd:  CAACAGCGACACCCACTCCT  GAPDH rev:  CACCCTGTTGCTGTAGCCAAA  HLTF fwd:  CGTATTAGAGAACCGGCCTTAC  HLTF rev:  GGATCACTCTTAGCCACCTTATG  ZRANB3 fwd:  GGTGTATGGTGGCTGATGAA  ZRANB3 rev:  AGAGACGAAGGGACCACTATTA  FANCL fwd: GGAGTGCAACAGCACGCAGAAT  FANCL rev: CTGCTCAGCTTAATTCCCAGGG RFWD3 fwd: ATCCGTGGACTGGCGTTTAGCA  RFWD3 rev: GCCTCATCAAGACACCAGCAAC PCNA fwd: CAAGTAATGTCGATAAAGAGGAGG  PCNA rev: GTGTCACCGTTGAAGAGAGTGG  ERCC1 fwd:  CTACAAGGCCTATGAGCAGAAA  ERCC1 rev:  CTTCACGGTGGTCAGACATT  ERCC4 fwd:  TGAAATTTACAGGGCGAGTAGG  ERCC4 rev:  TCCGCAAAGCAGTGAGATAG  GEN1 fwd:  ATGTTCCCATCCAGGTTCAC  GEN1 rev:  ACTCACAAGGACAGCAGTATTC  MUS81 fwd:  AGCTGCACGTTGGAGATTT  MUS81 rev:  CTCCACAATGTGATCCAGTACC  CHEK1 fwd:  GGTTGACTTCCGGCTTTCTAA  CHEK1 rev:  TCTTCTGGCTGCTCACAATATC  RPA1 fwd:  TCATCAACATCCGTCCCATTAC  RPA1 rev:  TCGCCAACATGAAAGAGGATAG  RAD9A fwd:  CCGCTCTTCTTCCAGCAATA  RAD9A rev:  CGGAAGACAGACAGGAAAGAC  BRCA1 fwd:  TCTGAAGACTGCTCAGGGCTAT  BRCA1 rev:  AGTTCAGCCATTTCCTGCTG |
| **Recombinant DNA** |  |  |
| pHR-UCOE-SFFV-Zim3-dCas9-P2A-Hygro | Marco Jost, Jonathan Weissman lab | Addgene plasmid # 188768 |
| pLVX-TetOne-Puro | Silva et al., 2019 | N/A |
| pLVX-TetOne-Puro-V5-FANCM_WT | Silva et al., 2019 | N/A |
| pLVX-TetOne-Puro-V5-FANCM_K117R | Silva et al., 2019 | N/A |
| pLVX-TetOne-Puro-V5-FANCM_ΔMM1 | This study | N/A |
| pLVX-TetOne-Puro-V5-FANCM_ΔMM2 | This study | N/A |
| pLVX-TetOne-Puro-3xFLAG-SMARCAL1_WT | This study | N/A |
| pLVX-TetOne-Puro-3xFLAG-SMARCAL1_R764Q | This study | N/A |
| pLVX-TetOne-Puro-3xFLAG-SMARCAL1_AA | This study | N/A |
| pLVX-TetOne-Puro-3xFLAG-SMARCAL1_ΔRBM | This study | N/A |
| pLVX-TetOne-Puro-3xFLAG-SMARCAL1_ΔHARP | This study | N/A |
| pLVX-TetOne-Puro-FEN1_WT | This study | N/A |
| pLVX-TetOne-Puro-FEN1_D181A | This study | N/A |
| pLVX-TetOne-Puro-LIG1_WT | This study | N/A |
| pLVX-TetOne-Puro-LIG1_K568G | This study | N/A |
| ppyCAG_RNaseH1_WT | Xiang-Dong Fu lab | Addgene plasmid #111906 |
| ppyCAG_RNaseH1_WKKD | Xiang-Dong Fu lab | Addgene plasmid #111905 |
| ppyCAG_RNaseH1_WT_IRES_mCherry | This study | N/A |
| ppyCAG_RNaseH1_WKKD_IRES_mCherry | This study | N/A |
| pRRlsinPGK_H2BGFP_WPRE | Beverly Torok-Storb lab | Addgene plasmid #91788 |
| pLenti-PGK-Neo-PIP-FUCCI | Jean Cook lab | Addgene plasmid #118616 |
| mTagRFP-T2-PCNA-19 | Michael Davidson lab | Addgene plasmid #58043 |
| pLVX-TetOne-Puro-PCNA_WT | This study | N/A |
| pLVX-TetOne-Puro-PCNA_K164R | This study | N/A |
| pShuttle-FEN1hWT | Sheila Stewart lab | Addgene plasmid #35027 |
| pDONR223_LIG1_WT_V5 | Jesse Boehm, Matthew Meyerson, David Root labs | Addgene plasmid #83006 |
| pAcGFP-C1-RPA123-P2A | Toledo et al., 2013 | N/A |
| pLVX-TetOne-Puro-RPA123-P2A | This study | N/A |
| pFB‐MBP‐SMARCAL1 | This study | N/A |
| pFB‐MBP‐SMARCAL1 ΔRBM | This study | N/A |
| pFB‐MBP‐FANCM | This study | N/A |
| pUC19_TA | Mengoli et al., 2023 | N/A |
| pUC19_chr3_16.54Mb | This study | N/A |
| **Software and Algorithms** |  |  |
| LI-COR ImageStudio software V5.2 | LI-COR | https://www.licor.com/bio/image-studio/ |
| Adobe Illustrator 2023 | Adobe Systems | https://creativecloud.adobe.com/en/apps/download/creative-cloud |
| Prism 9 | GraphPad | https://www.graphpad.com/scientific-software/prism/ |
| ImageJ | NIH | https://imagej.net/ij/index.html |
| QuickFigures ImageJ plugin | Mazo, 2021 | https://imagej.net/plugins/quickfigures |
| FlowJo_v10.8.1 | FlowJo | https://www.flowjo.com/solutions/flowjo/downloads |
| Inkscape 1.2.2 | Inkscape | https://inkscape.org/ |
| GEMINI | Zamanighomi et al., 2019 | https://github.com/sellerslab/gemini |
| Cytoscape 3.9.1 | Cytoscape | https://cytoscape.org/ |
| ICE CRISPR analysis tool | Synthego | https://www.synthego.com/products/bioinformatics/crispr-analysis |
| R 4.2.1/2 | R Core Team | https://www.R-project.org/ |
| R Studio | RStudio Team | http://www.rstudio.com/ |
